# ZIPK: A Unique Case of Murine-Specific Divergence of a Conserved Vertebrate Gene

**DOI:** 10.1371/journal.pgen.0030180

**Published:** 2007-10-19

**Authors:** Yishay Shoval, Shmuel Pietrokovski, Adi Kimchi

**Affiliations:** Department of Molecular Genetics, Weizmann Institute of Science, Rehovot, Israel; Lawrence Livermore National Laboratory, United States of America

## Abstract

Zipper interacting protein kinase (ZIPK, also known as death-associated protein kinase 3 [DAPK3]) is a Ser/Thr kinase that functions in programmed cell death. Since its identification eight years ago, contradictory findings regarding its intracellular localization and molecular mode of action have been reported, which may be attributed to unpredicted differences among the human and rodent orthologs. By aligning the sequences of all available ZIPK orthologs, from fish to human, we discovered that rat and mouse sequences are more diverged from the human ortholog relative to other, more distant, vertebrates. To test experimentally the outcome of this sequence divergence, we compared rat ZIPK to human ZIPK in the same cellular settings. We found that while ectopically expressed human ZIPK localized to the cytoplasm and induced membrane blebbing, rat ZIPK localized exclusively within nuclei, mainly to promyelocytic leukemia oncogenic bodies, and induced significantly lower levels of membrane blebbing. Among the unique murine (rat and mouse) sequence features, we found that a highly conserved phosphorylation site, previously shown to have an effect on the cellular localization of human ZIPK, is absent in murines but not in earlier diverging organisms. Recreating this phosphorylation site in rat ZIPK led to a significant reduction in its promyelocytic leukemia oncogenic body localization, yet did not confer full cytoplasmic localization. Additionally, we found that while rat ZIPK interacts with PAR-4 (also known as PAWR) very efficiently, human ZIPK fails to do so. This interaction has clear functional implications, as coexpression of PAR-4 with rat ZIPK caused nuclear to cytoplasm translocation and induced strong membrane blebbing, thus providing the murine protein a possible adaptive mechanism to compensate for its sequence divergence. We have also cloned zebrafish ZIPK and found that, like the human and unlike the murine orthologs, it localizes to the cytoplasm, and fails to bind the highly conserved PAR-4 protein. This further supports the hypothesis that murine ZIPK underwent specific divergence from a conserved consensus. In conclusion, we present a case of species-specific divergence occurring in a specific branch of the evolutionary tree, accompanied by the acquisition of a unique protein–protein interaction that enables conservation of cellular function.

## Author Summary

Mammals are a fairly young class of animals, first appearing about 70 million years ago. Such recent common descent does not allow the evolutionary process to create much diversity within the class, and indeed, the physiology among different mammals is remarkably similar. This similarity enables the use of various small mammals, especially rats and mice, as model systems for the study of biological phenomenon and disease. Experiments unfeasible or unethical to perform on humans are conducted on these model animals, with the postulation that insights gained from them are applicable to the human system. In this article, we present an exception to this rule. We bring evidence that ZIPK, a gene with important roles in programmed cell death, has undergone accelerated evolution in the rat and mouse, thus diverging considerably from a well-conserved consensus in all vertebrates, from fish to man. We also show that this sequence divergence caused changes in the protein's properties, including its localization within the cell, and the proteins with which it interacts. Still, the basic biologic function of ZIPK is conserved in both systems, and we propose an adaptive mechanism that compensates for the sequence divergence in rodents.

## Introduction

Orthologs are corresponding genes in different species. Such genes evolve from a common ancestral gene and usually have similar functions. The degree of divergence of orthologs can provide information on the taxonomic relationship between organisms and the similarity of their function. Orthologs from organisms that are closely related display a higher degree of similarity in the nucleotide sequence than distant organisms in the evolutionary tree. Here, we present an unusual exception to this rule in the ZIP-kinase genes.

Zipper interacting protein kinase (ZIPK, also called DAPK3 or DAPK like kinase (DLK)) is a member of the death associated protein kinase (DAPK) family. The family consists of a group of cell death-promoting Ser/Thr kinases homologous in their catalytic domains, including DAPK (DAPK1) and DRP-1 (DAPK2) [1]. Notably, the C-terminal extra-catalytic domains of these family members differ substantially from each other. In ZIPK, it comprises several putative nuclear localization signal (NLS) sequences and a leucine zipper domain, required for homo-oligomerization, interaction with other leucine zipper–containing proteins, and also critical for its death-promoting effects [2–5].

A long-standing debate exists in the literature concerning the intracellular localization of ZIPK, as well as its molecular mode of action. Several publications report that ectopic expression of catalytically active human ZIPK induces cell death, characterized by membrane blebbing and cell rounding [6]. These reports indicate that ZIPK is mostly localized to the cytoplasm, with a small fraction of cells also showing nuclear staining in a diffuse pattern. The catalytically inactive mutant ZIPK K42A was shown to localize exclusively to the cytoplasm, further suggesting that the kinase activity may have an impact on the intracellular localization [7]. In epithelial cell lines, ZIPK was shown to induce membrane blebbing through phosphorylation of the regulatory light chain of myosin II (MLC), and in some circumstances, to induce the formation of autophagic vesicles [6–8]. A second cytoplasmic function for ZIPK was observed in smooth muscle cells, where ZIPK-dependent phosphorylation of MLC led to Ca^+2^ sensitization and smooth muscle contraction. This was attributed to direct phosphorylation of MLC as well as inactivation of smooth muscle myosin phosphatase (SMMP-1M), through phosphorylation of the phosphatase's myosin binding subunit, and phosphorylation of its inhibitor protein CPI17 (PPP1R14A) [9,10].

In contrast, other studies from various researchers suggest a completely different mode of action and intracellular localization for ZIPK. It has been reported that ZIPK localizes predominantly to the nucleus, mostly appearing in a speckled staining pattern identified as promyelocytic leukemia oncogenic bodies (PODs) [2–4,11]. At the molecular level, ZIPK was shown by these groups to bind DAXX (also known as Fas death domain-associated protein 6) and thus regulate its recruitment to the PODs. ZIPK was also shown to interact with two transcription factors, ATF4, a member of the activating transcription factor/cyclic AMP-responsive element-binding protein (ATF/CREB) family and STAT3, a latent cytoplasmic transcription factor that can be activated by cytokines and growth factors [2]. Interestingly, one group reported that the prostate apoptosis response 4 (PAR-4), a substrate of ZIPK, is capable of translocating ZIPK from the PODs to the actin microfilaments [4,12,13].

Different arguments have been raised in the literature in an attempt to explain this basic discrepancy in localization/function, suggesting that differences in the type of cells or expression vectors used in these experiments accounted for the disparate results. Others argued that the differences were species specific, and depended on whether the human or mouse/rat orthologs were used in these studies [6,14]. Since the latter possibility has not been studied in a direct manner, we have undertaken integrated bioinformatics and experimental analyses of ZIPK orthologs to test this hypothesis.

Here, we report that through accelerated evolution of the ZIPK locus in the mouse and rat, these genes diverged considerably from the common consensus, highly conserved from fish to human. Among the various changes, the loss of a critical phosphorylation site that influences the protein's intracellular localization was identified. We also demonstrate that PAR-4/ZIPK interaction occurs in the murine system, but not in human or in the zebrafish ortholog, which, like the human ZIPK, also localizes to the cytoplasm. We suggest that the PAR-4–mediated cytoplasmic translocation in the murine system may provide a compensatory mechanism for conserving the membrane blebbing function of mouse and rat orthologs, an important feature in the mode of action of these kinases.

## Results/Discussion

### Murine ZIPK Has Diverged Considerably from the Conserved Consensus

**Figure 1:**
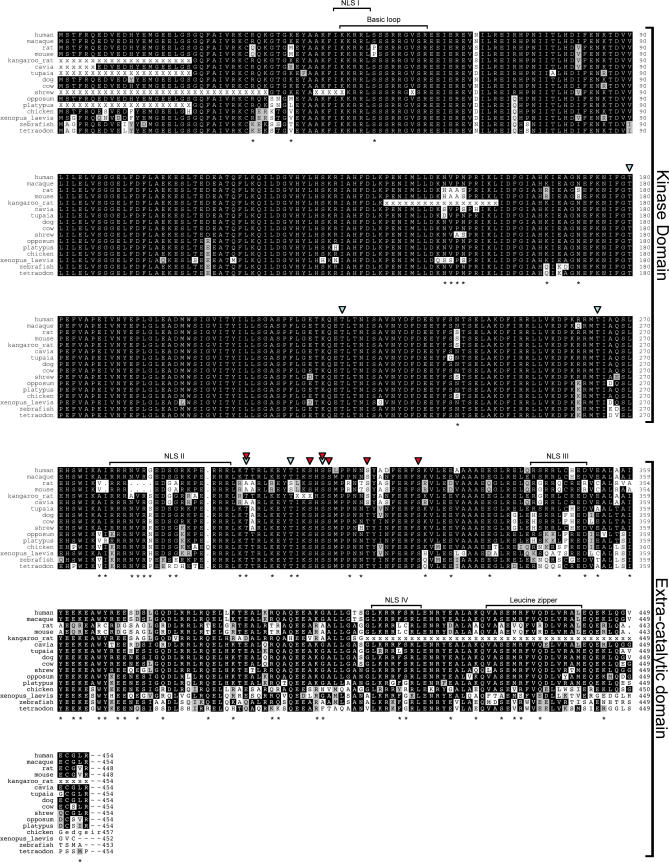
Multiple Sequence Alignment of the ZIPK Protein ZIPK protein sequences of the indicated organisms were extracted from the nonredundant database and from organism-specific genome projects, and aligned using the DIALIGN2 program. The alignment was corrected to allow the arginine triplet at position 278–280 of the rodent sequences to align with similar triplets in the other sequences. X's indicate unknown residues in partial sequences. Black shading indicates identity, grey shading indicates similarity. Asterisks mark sites of divergence of the rat and mouse orthologs from the mammalian consensus. Blue arrowheads indicate sites of autophosphorylation [5]; red arrowheads indicate sites of DAPK phosphorylation [8]. Putative NLSs I, II, and III, functional NLS IV, the basic loop, and the leucine zipper are indicated. Sequences of 16 representative organisms are displayed, representing fish, amphibians, birds, mammals, and, in particular, rodents. The full multiple sequence alignment of all ZIPK sequences used in this work is in Figures S1–S3.

**Figure 2:**
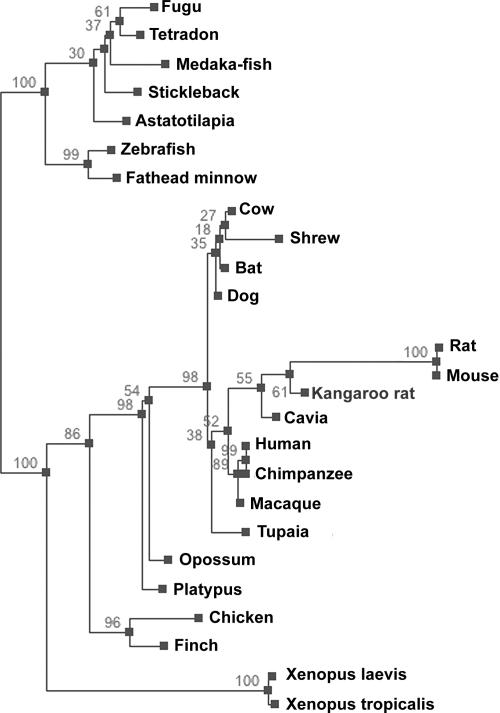
Phylogenetic Relationship of Vertebrates Based on ZIPK Proteins A phylogenetic tree of the indicated organisms was constructed based on the ZIPK multiple alignment, using the PHYML program. Numbers above branches represent bootstrap support from 100 replicates. Length of branches indicates extent of divergence.

The ZIPK gene is present in various vertebrates, from fish to mammals (Figures 1 and 2; accession numbers listed in Text S1), and its product is highly conserved in all the species in which it was found. Identities are at least 88% in the N-terminal kinase domain of ZIPK proteins (268 amino acids long), and at least 46% in the C-terminal regulatory domain (approximately 185 amino acids long), between fish and mammals (Figure 1). However, careful examination reveals that both mouse and rat ZIPK proteins are more divergent than expected. A dendogram computed from an alignment of ZIPK protein sequences shows that both rat and mouse sequences are on a long branch within the mammalian ZIPK protein cluster (Figure 2; the alignment of the protein sequences from the 25 species used to calculate the dendogram is shown in Figure S1, and the amino acid and DNA sequences are shown in Figures S2 and S3, respectively). Calculating the phylogenetic distance between the different ZIPKs shows that the rat and mouse sequences diverged from the human sequence to the same extent as chicken ZIPK, much further than expected. The dendogram has no other notable discrepancies from accepted taxonomy in either its topology or branch lengths. This finding was robust, being observed in all dendograms obtained using different organism sets and ZIPK regions (unpublished data). Most of the murine-unique sites (82%) appear in the C-terminal regulatory domain of the kinase (aa 269–454, according to the human nomenclature). Altogether, the murine C-terminal region contains 47 unique sites, as compared to the mammalian consensus, corresponding to 25% of the sequence.

The rat and mouse sequences are very conserved between themselves, having only one nonidentical position (Val/Ala at aa 358). The two other rodents whose ZIPK sequence we found, *Cavia* (guinea pig) and kangaroo rat, possess only a small portion of the murine unique sites, and are more similar to other mammals (Figure 1). In the dendogram, they are grouped together with the rat and mouse to form the group of rodents, but their shorter branch length indicates that they have diverged from the mammalian consensus to a lesser extent (Figure 2). The shrew, an even smaller mammal, which also has a short life span and numerous progeny, does not show extended branch length on the dendogram (Figure 2). Thus, it is unlikely that the extent of the divergence we found in the murine ZIPK sequences emerged from a more general process of accelerated evolution due to short life span and numerous progeny of murines. Additionally, two other proteins, which were examined in a similar manner, did not show exceptional murine branch lengths. These were PAR-4 (a protein interacting with ZIPK, see below) and DAPK (a ZIPK family member sharing some similar functions due to a common kinase domain and capable of interacting and transphosphorylating ZIPK [8]) proteins (Figures S4 and S5 for DAPK and PAR-4, respectively). A large-scale analysis of mammalian evolution also found that the rate of molecular evolution in rodent and carnivore lineages is the same and is ∼11%–14% faster than in the primate lineage [15]. Other works also did not find a particularly hyper-accelerated evolution of murines, such as what we found for ZIPKs [16–18]. The divergence therefore appears to be specific to the ZIPK gene and to the murine system.

To understand the significance of the changes in the protein sequence of rat and mouse ZIPK, we superimposed on the ZIPK multiple sequence alignment the functional data generated in our lab and others regarding human, rat, and mouse ZIPK proteins (Figure 1), and examined which of the murine unique sites lie in these functional domains. Functional annotations included the potential NLSs, the leucine zipper domain, and Ser/Thr residues previously shown to be targets for autophosphorylation and/or *trans*-phosphorylation by DAPK.

The kinase domain contains only two notable divergences in the rat and mouse ZIPK: a serine to proline substitution at position 50 located in a basic loop within the catalytic domain, and a 4-aa substitution at positions 151–154 (NVPN to HAAS). The basic loop (aa 45–57, mostly positively charged), known as the fingerprint of the DAP kinase family of proteins, is exposed on the surface of the catalytic domain, as shown in the crystal structure of another member, DAPK [19]. Previous work has indicated that the basic loops of human ZIPK and DAPK are essential for a physical interaction between the two kinases, a process followed by *trans*-phosphorylation and amplification of the death signals [8]. Yet, we found that the physical interaction between the catalytic domain of DAPK and the full-length rat ZIPK was similar to that of human ZIPK/DAPK interaction, suggesting that the serine-to-proline substitution had no effect on the binding property (unpublished data). Recently, though, it has been suggested that serine 50 in human ZIPK undergoes autophosphorylation (A. P. Turnbull et al., Protein Data Bank entry 2J90; http://www.rcsb.org/pdb/explore/explore.do?structureId=2J90). The putative functional implications of this phosphorylation would be absent in the murine ZIPK proteins, a point to be addressed in the future. The 4-aa substitution at positions 151–154 falls within another loop, between helices VI and VII of the kinase, and its significance is, as yet, unknown.

In the extra-catalytic part of the protein, the most apparent change in murine ZIPK is a 5-aa deletion (between position 277–284 in human), which falls within a putative bipartite NLS, previously classified as NLS II [4]. The rat and mouse ZIPK proteins have lost part of the linker region between the positively charged ends of this potential NLS. Yet, in light of previous work showing that NLS IV, at the C terminus of the protein (position 409–416 of the human protein), is the functionally relevant NLS, required and sufficient for nuclear localization of murine ZIPK [4], the possible functional implication of the loss of the linker region in NLS II in influencing the nuclear localization of ZIPK is unclear. NLS IV contains two murine substitution sites, but the effect of these substitutions is predicted to be minor, as all of the positively charged amino acids important for the nuclear localization signal are conserved. The leucine zipper structure, at the C-terminal part of the extra catalytic domain, is conserved between human and rodents, as the heptad Leu/Val repeats at positions 427/434/441, previously shown to compose the leucine zipper [2], are unchanged.

Another site of significant divergence noted among the functionally relevant regions in the protein relates to phosphorylation sites on ZIPK. Human ZIPK was previously shown to undergo both autophosphorylation as well as *trans*-phosphorylation by DAPK at multiple sites (Figure 1) [5,8]. These phosphorylation events, two of which result from both auto- and *trans*-phosphorylation, have been shown to have prominent functional effects on either the catalytic activity or the cellular localization of human ZIPK. As human ZIPK and DAPK share several common substrates, it has been shown that the *trans*-phosphorylation by DAPK, which occurs following the physical interaction between the two kinases, creates a feed-forward regulatory process leading to amplification of the cell death–promoting signals [8]. Examining the conservation of these sites revealed that most are conserved in murine ZIPK. The only nonconserved phosphorylation site is threonine 299, a site that was identified as a common target of both auto- and *trans*-phosphorylation [5,8]. The phosphorylation of threonines 299–300 was shown to have an impact on the cellular localization of human ZIPK; ZIPK bearing the T299A/T300A phospho-silencing mutation is mainly nuclear, while the T299D/T300D phospho-mimicking mutant is a mainly cytoplasmic form [5]. The murine ZIPK protein thus lacks a critical phosphorylation site that affects cellular localization.

Notably, rat, mouse, and *Cavia* ZIPKs have alanines in positions 299 and 300, while the kangaroo rat, the other rodent ZIPK sequence found, has the conserved threonines at these positions. Within the rodents, the kangaroo rat, rat, and mouse are in a different cluster than the cavia [16]. This indicates either convergent mutations in the rat/mouse and cavia, or a reversion in the kangaroo rat.

### The Rat ZIPK Differs in Its Intracellular Localization from Human and Zebrafish Orthologs

**Figure 3:**
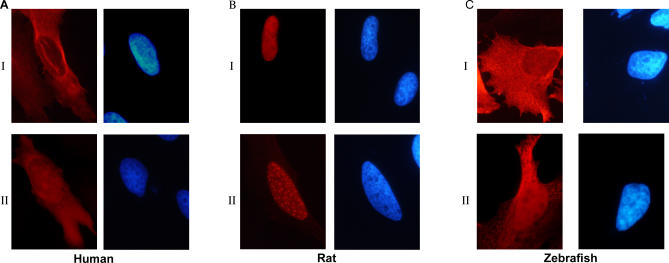
Rat ZIPK Shows Different Cellular Localization from Human and Zebrafish ZIPK Cellular localization of ectopically expressed FLAG-human ZIPK (A), FLAG-rat ZIPK (B), and FLAG-zebrafish ZIPK (C) in human HeLa cells, expressed at sublethal concentrations. Immunostaining was preformed using anti-FLAG antibodies. Shown are the two classes of localization observed for each species. In (A) and (C): I, cytoplasmic staining excluded from nuclei; II, nuclear and cytoplasmic. In (B): I, diffuse nuclear; II, punctate nuclear, previously shown to colocalize with promyelocytic leukemia bodies.

As discussed above, there are conflicting data in the literature concerning the intracellular localization of ZIPK protein. In order to directly test whether this reflects differences between species, we expressed either human or rat FLAG-tagged ZIPK in human HeLa cells, at sublethal concentrations, and followed their intracellular localization by immunostaining. Rat ZIPK was localized to the nucleus, showing in 50%–60% of the cells a punctate nuclear staining that was previously defined as being associated with PODs (Figure 3BI and 3BII for diffuse and punctate nuclear staining, respectively). The human ortholog was mainly cytoplasmic and was excluded from nuclei (Figure 3AI), with a small fraction also showing a diffuse nuclear staining, yet with no punctate staining at all (Figure 3AII). We repeated the experiment using GFP-conjugated proteins, with similar results (unpublished data). To our knowledge, this is the first time the localization of both human and rat ZIPK was examined and compared in the same cells with the same vectors, and thus it is clear that the differences in localization stem from the proteins themselves.

To determine whether the difference in cellular localization was due to the specific divergence of murine ZIPK from a common evolutionarily conserved consensus, FLAG-tagged zebrafish ZIPK was cloned and expressed in the same cellular setting described above. Zebrafish ZIPK immunostaining was very similar to human ZIPK, localizing mostly to the cytoplasm (Figure 3CI) with a small percentage showing diffused nuclear staining in addition to the cytoplasmic staining (Figure 3CII).

### A299T/A300T Mutation Changes the Pattern of Nuclear Localization and Slightly Enhances the Blebbing Potency of Rat ZIPK

**Figure 4:**
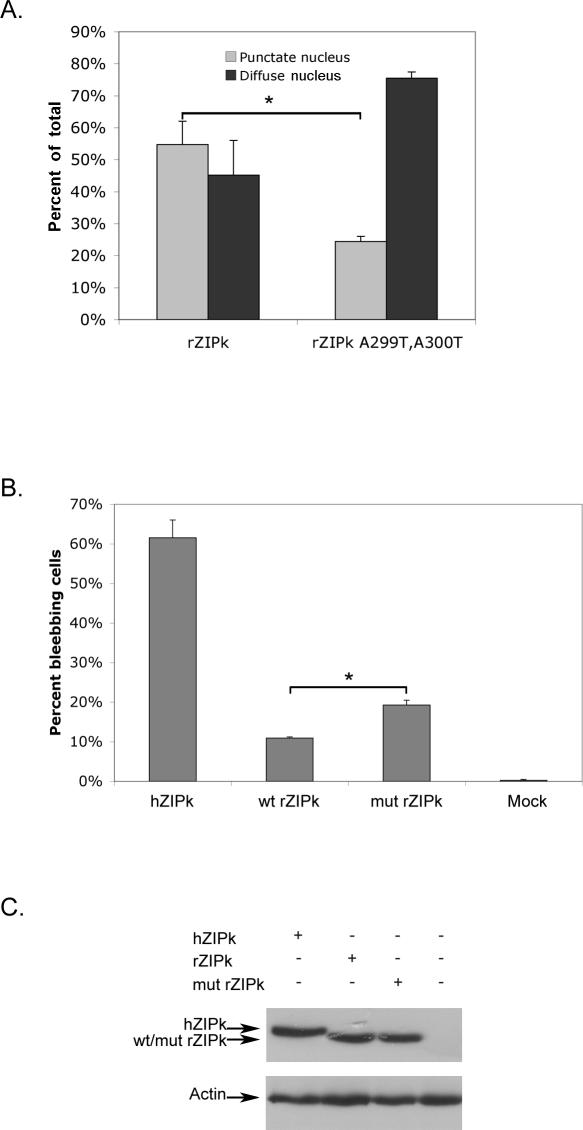
A299T/A300T Mutation in Rat ZIPK Causes Dissociation from PODs and Increased Blebbing-Inducing Potency (A) Cellular localization of ectopically expressed wild-type and mutant (A299T and A300T) rat ZIPK in HeLa cells. Quantities are the means of three independent experiments; 100 cells were counted in each experiment, asterisk denotes *p* < 0.005. (B). Blebbing-inducing capacity of ectopically expressed human ZIPK, wild-type, and mutant (A299T and A300T) rat ZIPK in HeLa cells. The percent of blebbing cells out of GFP-positive cells was determined by counting 100 cells. Quantities are the means of three independent experiments, asterisk denotes *p* < 0.0005. (C) Western blot showing equal expression levels of the ectopically expressed proteins.

To check whether the loss of the phosphorylation sites at positions 299 and 300 in rat ZIPK might influence murine protein nuclear localization, we generated an A299T/A300T rat ZIPK mutant, thus recreating the phosphorylation sites. Comparing the cellular localization of ectopically expressed wild type and mutant rat ZIPK in HeLa cells revealed that the localization of the protein changed in a specific manner (Figure 4A). Both wild-type and mutant rat ZIPK are still exclusively nuclear, but whereas the wild type is mostly associated with PODs, the mutant rat ZIPK is mostly nucleoplasmic. Thus, it seems that the A299T/A300T mutation reduced the localization to PODs characteristic of the murine orthologs, yet was not sufficient by itself to impose a massive translocation to the cytoplasm.

A small yet functionally significant fraction of the rat ZIPK mutant molecules, which have lost the POD staining, might translocate to the cytoplasm while still remaining below the immunostaining threshold sensitivity. To approach this possibility we turned to a more sensitive assay that measures the membrane blebbing effects of ZIPK, previously shown to be caused by MLC phosphorylation on microfilaments. We compared the membrane blebbing potency of wild type and A299T/A300T rat ZIPK, and of human ZIPK. As shown in Figure 4B, mutant rat ZIPK is more potent in the induction of blebbing than wild-type rat ZIPK, although not as potent as human ZIPK. All the tested ZIPK proteins were expressed to the same extent in these experiments (Figure 4C). To examine more carefully the intracellular localization of mutant ZIPK in the blebbing cells we turned to HEK 293 cells, in which the blebbing phenotype is more pronounced, and ectopically expressed proteins reach higher levels. In some of the blebbing cells, we could detect the mutant rat ZIPK within the bleb structures, suggesting that some nuclear-to-cytoplasm translocation takes place in the mutant rat ZIPK (Figure S6).

Altogether, the small yet statistically significant increase in blebbing capacity and the dissociation from PODs in the A299T/A300T mutant indicate that the loss of the phosphorylation sites at aa 299 and 300 in the rat and mouse may partially explain the phenotypic differences between the human and murine proteins. Still, the blebbing-inducing capacity of mutant rat ZIPK is significantly lower than that of human ZIPK, suggesting that additional sequence changes may be involved, and are most probably part of the other unique structural differences discussed above.

### PAR-4, a Murine ZIPK Interacting Protein, Does Not Bind the Human or Zebrafish ZIPK Orthologs

**Figure 5:**
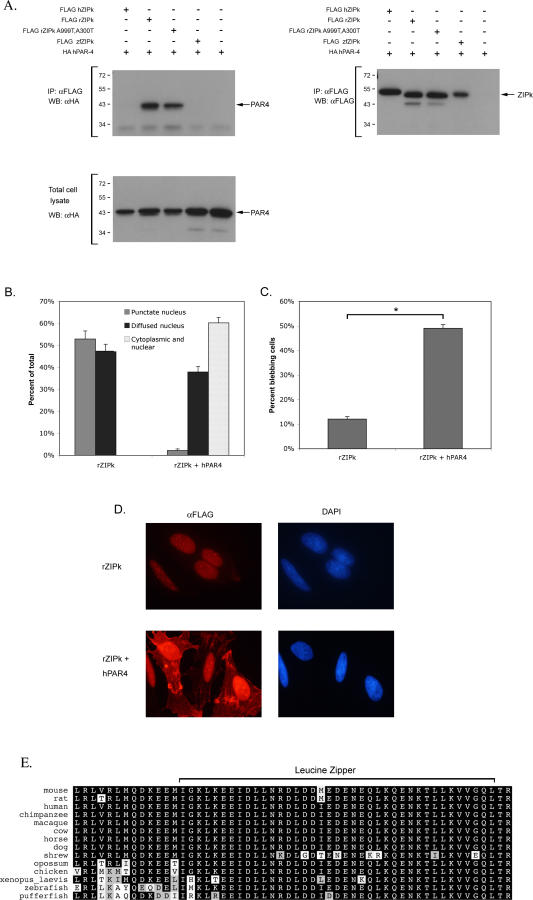
PAR-4 Binds Rat, but Not Human or Zebrafish, ZIPK (A) HA-tagged human PAR-4 protein was coexpressed with FLAG-tagged human ZIPK, wild-type rat ZIPK, A299T/A300T rat ZIPK, or zebrafish ZIPK in 293 cells. Proteins were immunoprecipitated using anti-FLAG antibodies and the resulting immunoprecipitates were probed with anti-HA (left) or anti-FLAG (right) antibodies. Blot of total cell lysates shows equal amounts of PAR-4 expression in the different coexpression assays. (B) Quantification of the cellular localization of rat ZIPK alone and coexpressed with human PAR-4 in HeLa cells. The percent of cells displaying one of the three localization profiles was determined by counting 100 cells in each slide. Quantities are the means of three independent experiments. (C) Blebbing-inducing capacity of ectopically expressed rat ZIPK alone and coexpressed with human PAR-4 in HeLa cells. Quantities are the means of three independent experiments, asterisk denotes *p* < 0.005. (D) Cellular localization of ectopically expressed rat ZIPK alone and coexpressed with human PAR-4. HeLa cells were immunostained using anti FLAG antibodies. (E) Multiple sequence alignment of the C-terminal part of PAR4. Protein sequences of the indicated organisms were extracted from the nonredundant database and aligned using the DIALIGN2 program. The leucine zipper is indicated.

The observed significant differences in the intracellular localization of the wild-type ZIPK proteins suggest that the human and murine ZIPK reside in different microenvironments, accessible to different interacting proteins and probably subjected to different regulatory mechanisms. This raises the question of how the cellular function of these divergent proteins has been conserved in spite of these changes. We therefore searched for an adaptive mechanism exclusive to the murine system that may allow the nuclear murine ZIPK to exit the nucleus and perform its cytoplasmic functions, thus compensating for the sequence divergence. Previous studies have shown that rat and mouse ZIPK both bind PAR-4 protein [4,11,13]. PAR-4 contains nuclear localization and export sequences, and is also able to bind actin microfilaments (MF). Upon overexpression, rat PAR-4 was shown by most studies to shuttle rat ZIPK from the PODs in the nucleus to the MFs, where ZIPK phosphorylates MLC and induces membrane blebbing [12,13]. The ability of PAR-4 to bind rat ZIPK, its ability to bind actin MF, and the actual ZIPK catalytic activity were all shown to be necessary for the induction of this phenotype. It therefore became of interest to test whether the PAR-4 binding capacity is unique to the murine system. To this end, we examined whether the two other orthologs, human and zebrafish ZIPK, also interact with PAR-4. We also examined in these experiments the possible influence of the mutations in position 299 and 300 of the rat ZIPK on PAR-4 binding. FLAG-tagged human ZIPK, wild-type, and A299T/A300T mutant rat ZIPK, and zebrafish ZIPK proteins were coexpressed with HA-tagged PAR-4 in HEK 293 cells, and ZIPK proteins were immunoprecipitated and assessed for their ability to pull down PAR-4. We used the human PAR-4 ortholog for these binding experiments in light of the high degree of conservation of PAR-4 in evolution (see Figure S7 for full sequence alignment), and considering the fact that the most critical question was to study whether ZIPK/PAR-4 interactions exist in the human system. The similarity between PAR-4 orthologs is extremely high, especially along the region of PAR-4 that binds to ZIPK (this region corresponds to the leucine zipper domain of PAR-4 spanning between aa 279–332 [20]), and, as shown in the alignment in Figure 5E, this domain displays 98% and 94% similarity between human PAR-4 and rat or zebrafish orthologs, respectively. Thus, it was not surprising to learn that human PAR-4 strongly interacted with the rat ZIPK. Also the mutations at position 299/300 in the rat ZIPK did not interfere with this binding (Figure 5A). Furthermore, PAR-4 coexpression displayed very pronounced effects on both the cellular localization and the membrane blebbing capacity of rat ZIPK, consistent with previous reports. As shown in Figure 5B and 5D, rat ZIPK translocated to the cytoplasm upon coexpression with PAR-4. Scoring the transfected cells for membrane blebbing indicated that rat ZIPK induced massive membrane blebbing when co-expressed with PAR-4, yielding values which approached those obtained upon ectopic expression of human ZIPK alone (Figure 5C). In contrast, we found that neither human nor zebrafish ZIPK could pull down human PAR-4 (Figure 5A). Although the PAR-4 binding region in ZIPK has not been well defined, it notably involves regions from the C-terminal part of the kinase (between aa 337–417 [13] or between aa 397–448 [11]). Our experimental data therefore suggests that the overall high sequence divergence within these C-terminal regions of murine ZIPK (Figure 1) dictates the PAR-4 interaction.

This result, along with the differences in localization, proves that the divergence of the murine ZIPK sequence from the common conserved consensus changes the biochemical properties of the protein relative to that of human or zebrafish ZIPK. We suggest that the interaction between murine ZIPK and PAR-4 evolved as a mechanism to compensate for the localization of ZIPK to the PODs by exporting it from the PODs in the nucleus to the cytoplasm, where it can fulfill one of the major functions of ZIPK, which is membrane blebbing.

To conclude, we present here an interesting case in which a highly conserved gene in all vertebrates has diverged considerably and specifically in the murine lineage. We show that murine ZIPK protein differs significantly from its human ortholog, losing an important auto- and *trans*-phosphorylation site and displaying distinct altered cellular localization. All this affects the regulation and, possibly, the activity of murine ZIPK. Yet, a different protein interaction capacity, with an important protein partner in the apoptosis pathway, evolved in the murine system to maintain the basic membrane blebbing function of these kinases.

Research on mouse and rat proteins in order to gain insights into the function of their human orthologs is widespread. The common conviction is that these three mammalian systems are close enough in evolution to make these deductions valid. Many studies have demonstrated that human and murine orthologs act and are regulated in the same manner. Here, we present an extraordinary exception to that rule, where data learned in one system could not be fully projected onto the other. Hence, the possibility of divergence and dissimilarity should be kept in mind when transferring knowledge even between seemingly closely related organisms. Still, the basic rule of functional conservation is valid through circumventing mechanisms.

## Materials and Methods

### Sequences.

Accession numbers for the ZIPK sequences used are in the Accession Numbers list under Supporting Information. ZIPK sequences extracted by us from genomic contigs, ESTs, or assembled raw sequencing traces (the latter indicated by TI), are available from the National Center for Biotechnology Information (NCBI) trace archive database and are listed in the Accession Numbers section. Assembly was done with the CAP3 program [21]. When needed, sequences from different sources for the same organism were combined. The coding regions on the genomic contigs are detailed in Figure S3.

### Multiple sequence alignments and phylogenetic tree.

Multiple sequence alignments were generated using the DIALIGN2 program [22]. ZIPK alignments were corrected to allow the arginine triplet at position 278–280 of the rodent sequences to align with similar triplets in the other sequences; this change was evident using other alignment programs (unpublished data). Sequence dendograms were calculated based on the full-length ZIPK multiple alignment, and the indicated PAR-4 and DAPK alignments, using the PHYML v.2.4.4 program [23].

### Plasmids.

Human ZIPK plasmid was previously described [8]. GFP-conjugated rat ZIPK was kindly provided by Karl Heinz Scheidtmann, from which the rat ZIPK (unfused to GFP) was subcloned into a FLAG-tagged pcDNA3 expression plasmid. A299T/A300T mutations were generated by PCR-mediated site-directed mutagenesis, using the QuikChange kit from STRATAGENE, following the provided protocol. All mutations were confirmed by direct sequencing. Human PAR-4 cDNA clone was purchased from RZPD, the German Resource Center for Genome Research, Berlin, Germany (I.M.A.G.E. Consortium [LLNL] cDNA clones) (clone ID IRAUp969D0743D6) and subcloned into an HA-tagged pcDNA3 expression plasmid. Zebrafish ZIPK was cloned from a 48-h embryo cDNA library, kindly provided by Nataliya Borodovsky and Gil Levkowitz, into a FLAG-tagged pcDNA3 expression plasmid.

### Cell culture and transient transfection.

293 Human Embryonic Kidney cells and HeLa cells were grown in DMEM (Biological Industries) supplemented with 10% fetal bovine serum (Hyclone) and 1% L-glutamine (GibcoBRL) and a mixture of antibiotics (100 U/ml penicillin and 0.1 mg/ml streptomycin). For transient transfections, 1.2 × 10^6^ (293) or 0.8 × 10^6^ (HeLa) cells were plated on 9-cm plates 24-h prior to transfection. Transfections were done by the calcium phosphate method with 10 μg DNA per plate. To assess the membrane-blebbing potency of ZIPK, HeLa cells were transfected with 9 μg of the appropriate ZIPK construct and 1 μg of peGFP expression vector. After 24 h, green cells were counted and the percent of blebbing cells was calculated.

### Immunoprecipitation and Western blotting.

Cells were washed twice in PBS and then suspended in cold lysis buffer (20 mM Tris pH 7.5, 0.5% NP-40, 150 mM NaCl) with protease inhibitors (1% protease inhibitor cocktail [Sigma], 1% PMSF) and passed through a 21-gauge needle 20 times to break nuclei. Lysates were centrifuged for 15 min at 14,000 rpm at 4 °C. The pellet was discarded and the supernatant was precleared for 1 h at 4 °C on a slurry of protein G-PLUS Agarose beads (Santa Cruz Biotechnology). The precleared extracts were incubated with agarose-conjugated anti-FLAG M2 gel beads (Sigma) for 2 h at 4 °C. Immunoprecipitates were washed four times with lysis buffer containing protease inhibitors, and resolved by standard SDS-PAGE. Blots were reacted with anti-FLAG-M2 monoclonal antibody (dilution 1:500) (Sigma); anti-FLAG polyclonal antibody (dilution 1:800) (Sigma); anti-HA monoclonal antibody (dilution 1:1000) (Babco); or anti-Actin monoclonal antibody (dilution 1:5000) (Sigma).

### Immunostaining.

HeLa cells (0.8 × 10^6^)were seeded on glass cover slips in 9-cm plates and transfected the next day with the appropriate constructs, 10 μg DNA per plate. After 24 h, cells were fixed in 3.7% formaldehyde for 15 min. After blocking and permeabilization with 10% normal goat serum (Biological Industries), 0.4% Triton X-100 in PBS, the cells were incubated for 1 h with anti-FLAG polyclonal antibody (Sigma; 1:600 dilution) followed by RRX-conjugated goat anti-rabbit secondary antibody (Jackson ImmunoResearch; dilution 1:800). The cover slips were finally stained with DAPI (0.5 μg/ml, Sigma) and mounted with ImmuMount (Thermo Shandon) embedding media. Stained cells were viewed by fluorescent microscopy (Olympus BX41) equipped with a 100× oil immersion objective, using excitation wavelengths of 530–550 nm (for RRX) and 360–370 nm (for DAPI). Digital imaging was performed with a DP50 CCD camera using Viewfinder Lite and Studio Lite software (Olympus). Final composites were prepared in Adobe Photoshop (Adobe Systems).

## Supporting Information

Figure S1 Full Multiple Sequence Alignment of the ZIPK Protein ZIPK protein sequences of the indicated organisms were extracted from the nonredundant database and from organism-specific genome projects, and aligned using the DIALIGN2 program. The alignment was corrected to allow the arginine triplet at position 278–280 of the rodent sequences to align with similar triplets in the other sequences. X's indicate unknown residues in some partial sequences. Black shading indicates identity, grey shading indicates similarity.
(2.1 MB PDF)

Figure S2 ZIPK Protein Sequences ZIPK protein sequences of the indicated organisms were extracted from the nonredundant database and from organism-specific genome projects. X's indicate unknown residues in partial sequences.
(84 KB DOC)

Figure S3 ZIPK Protein Coding Sequences ZIPK protein coding regions of the indicated organisms were extracted from the NCBI databases (nr, EST, traces [TDB]) and from organism-specific genome projects. N's indicate unknown residues in partial sequences.
(56 KB PDF)

Figure S4 Phylogenetic Relationship of Vertebrates Based on DAPK Protein A dendrogram of the indicated organisms was calculated based on a DAPK multiple alignment, using the PHYML program. Numbers above branches represent bootstrap support from 100 replicates. Length of branches indicates extent of divergence. The tree is rooted by the position of invertebrate DAPK (e.g., nematode).
(570 KB TIF)

Figure S5 Phylogenetic Relationship of Vertebrates Based on PAR-4 Proteins A dendogram of the indicated organisms was calculated based on the PAR-4 multiple alignment shown in Figure S6, using the PHYML program. Numbers above branches represent bootstrap support from 100 replicates. Length of branches indicates extent of divergence.
(3.4 MB TIF)

Figure S6 Multiple Sequence Alignment of the PAR-4 Protein PAR-4 protein sequences of the indicated organisms were extracted from the nonredundant database, and aligned using the MACAW program [24]. Only uppercase regions are considered aligned. The underlined regions were used to construct the dendogram in Figure S5. aa 279–332 in the mouse and rat, containing the leucine zipper, were shown to bind ZIPK, and are highly conserved (Figure 5E).
(37 KB DOC)

Figure S7 Rat ZIPK Localization in Spread and Blebbing HEK 293 Cells Cellular localization of ectopically expressed FLAG.A299T/A300T rat ZIPK in human HEK 293 cells. Red, anti FLAG staining; blue. DAPI nuclear staining; green, GPF fluorescence. I. Spread cell, showing nuclear staining. II. Blebbing cell, showing staining in the blebs. Immunostaining was preformed using anti-FLAG antibodies.
(5.7 MB TIF)

Text S1 Accession Numbers (40 KB DOC)

### Accession Numbers

The NCBI Entrez (http://www.ncbi.nlm.nih.gov/sites/gquery) accession numbers for the ZIPK sequences used in this paper are *Bos taurus,* 76622257; *Canis familiaris,* 73987437; *Danio rerio,* 68356496; *Homo sapiens,* 4557511; *Macaca mulatta,* 109122939; *Mus musculus,* 6681133; *Pan troglodytes,* 114674687; *Rattus norvegicus,* 11968142; *Tetraodon nigroviridis,* 47223108; *Xenopus laevis,* 66911521; and *Xenopus tropicalis,* 62860094

The ZIPK sequences extracted by us from genomic contigs, ESTs, or assembled raw sequencing traces (the latter indicated by TI and available from the NCBI trace archive database (http://www.ncbi.nlm.nih.gov/Traces) under NCBI accession numbers Astatotilapia burtoni, 46489038; Cavia porcellus, TI|(812701167 + 816150362) + TI|(1589066801 + 816143923 + 1588856408 + 812549142 + 1586592910 + 760990457 + 1593835765 + 1587132918) + TI|1583699030 + 91731898; Dipodomys ordii, TI|(1542302641 + 1541211393 + 1578971935 + 1562912636 + 1552706462) + TI|(1569549916 + 1566271599 + 1585076393 + 1585061768 + 1542035913 + 1556201828 + 1541200922 + 1586154871 + 1585648556 + 1535177412) + TI|1587085697 + TI|1569544450; Fugu rubripes, 22419072; Gallus gallus, 51039807 + 25936497 + 25365707 +TI|227294367; Gasterosteus aculeatus, 86297297; Monodelphis domestica, 84819837; Myotis lucifugus, TI|(976759098 + 964301557 + 981846460) + 105860175; Ornithorhynchus anatinus, 91473547; Oryzias latipes, 16991872; Pimephales promelas, 72422020 + 73556779 + 73634852 + 73512480 + 73721434 + 72761900 + 73439047 + 73721433; Sorex araneus, 80402909 + 80402905 + TI|(838222606 + 892417228) + TI|(873967520 + 875830371 + 894062723 + 845045749 + 848969792); Taeniopygia guttata, TI|(1401310278 + 1277343563 + 1253220430 + 1397767862 + 1249786453 + 1290200497 + 1398826774 + 1397636686 + 1423694077) + TI|(1242613131 + 1422732028 + 1404775456 + 127731875 1) + TI|1241607171; and Tupaia belangeri, 108226212 + 107812812.

## Abbreviations

DAPK: death associated protein kinase
MF: microfilament
MLC: myosin light chain
NLS: nuclear localization signal
PAR-4: prostate apoptosis response 4
PODs: promyelocytic leukemia oncogenic bodies
ZIPK: zipper interacting protein kinase
