# Supplementary material for: ZIPK: A Unique Case of Murine-Specific Divergence of a Conserved Vertebrate Gene
Source: PLoS Genet. 2007 Oct 19;3(10):e180. doi: 10.1371/journal.pgen.0030180 (PMC2041995; doi:10.1371/journal.pgen.0030180)
Supplement: Supplementary file 1 [file pgen.0030180.sg001.pdf]

Fig. S1

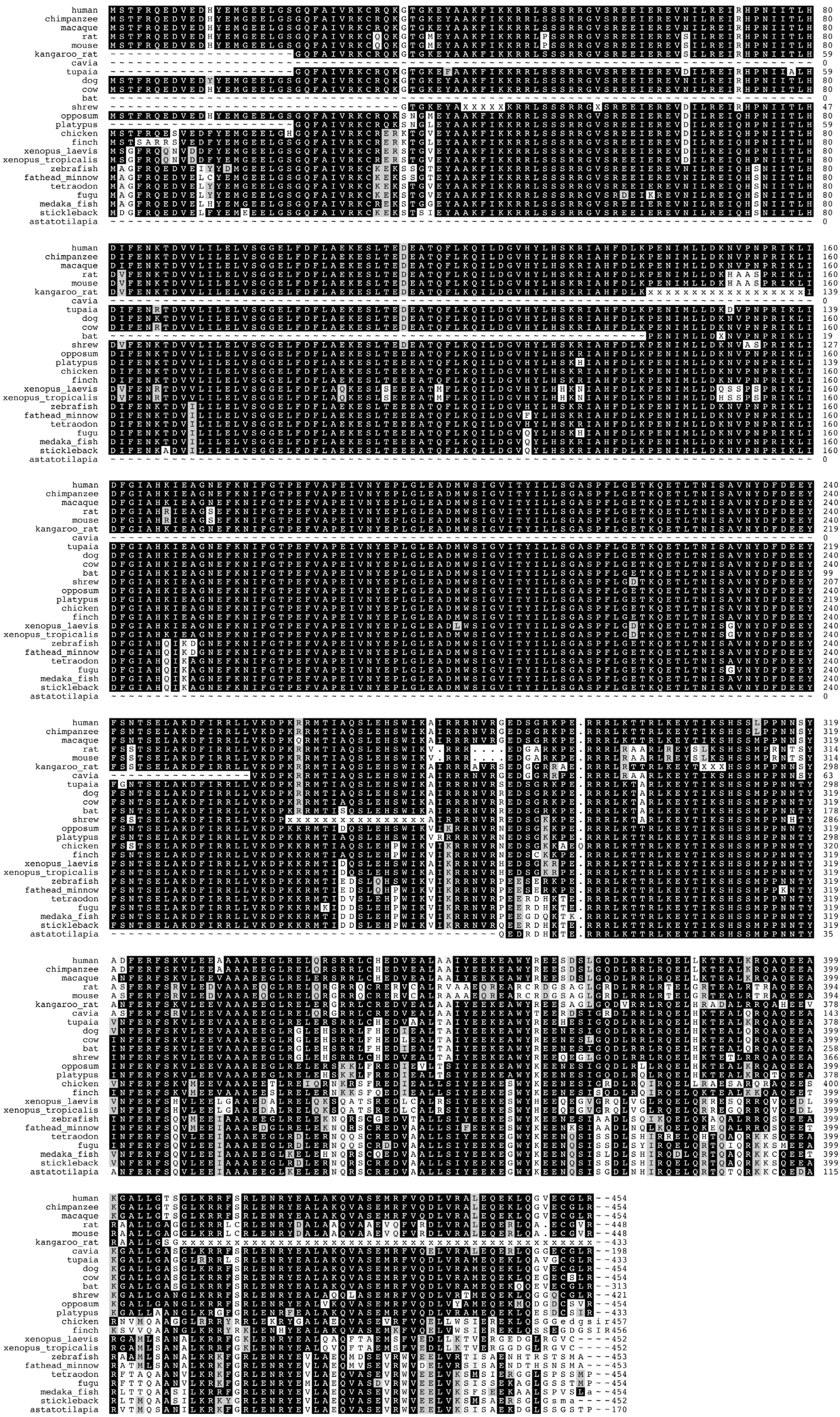
human
chimpanzee
macaque
rat
mouse
kangaroo_rat
cavia
tupaia
dog
cow
bat
shrew
opposum
platypus
chicken
finch
xenopus_laevis
xenopus_tropicalis
zebrafish
fathead_minnow
tetraodon
fugu
medaka_fish
stickleback
astatotilapia
