# Supplementary material for: ZIPK: A Unique Case of Murine-Specific Divergence of a Conserved Vertebrate Gene
Source: PLoS Genet. 2007 Oct 19;3(10):e180. doi: 10.1371/journal.pgen.0030180 (PMC2041995; doi:10.1371/journal.pgen.0030180)
Supplement: Supplementary file 2 [file pgen.0030180.sg003.pdf]

Fig. S3

>dapk3_human gi|2911155:94-1458 Homo sapiens mRNA for ZIP-kinase, complete cds, 1365 bases, A83C11AD checksum.
ATGTCCACGTTCAGGCAGGAGGACGTGGAGGACCATTATGAGATGGGGGA
GGAGCTGGGCAGCGGCCAGTTTGCGATCGTGCGGAAGTGCCGGCAGAAGG
GCACGGGCAAGGAGTACGCAGCCAAGTTCATCAAGAAGCGCCGCCTGTCA
TCCAGCCGGCGTGGGGTGAGCCGGGAGGAGATCGAGCGGGAGGTGAACAT
CCTGCGGGAGATCCGGCACCCCAACATCATCACCCTGCACGACATCTTCG
AGAACAAGACGGACGTGGTCCTCATCCTGGAGCTGGTCTCTGGCGGGGAG
CTCTTTGACTTCCTGGCGGAGAAAGAGTCGCTGACGGAGGACGAGGCCAC
CCAGTTCCTCAAGCAGATCCTGGACGGCGTTCACTACCTGCACTCTAAGC
GCATCGCACACTTTGACCTGAAGCCGGAAAACATCATGCTGCTGGACAAG
AACGTGCCCAACCCACGAATCAAGCTCATCGACTTCGGCATCGCGCACAA
GATCGAGGCGGGGAACGAGTTCAAGAACATCTTCGGCACCCCGGAGTTTG
TGGCCCCAGAGATTGTGAACTATGAGCCGCTGGGCCTGGAGGCGGACATG
TGGAGCATCGGTGTCATCACCTATATCCTCCTGAGCGGTGCATCCCCGTT
CCTGGGCGAGACCAAGCAGGAGACGCTCACCAACATCTCAGCCGTGAACT
ACGACTTCGACGAGGAGTACTTCAGCAACACCAGCGAGCTGGCCAAGGAC
TTCATTCGCCGGCTGCTCGTCAAAGATCCCAAGCGGAGAATGACCATTGC
CCAGAGCCTGGAACATTCCTGGATTAAGGCGATCCGGCGGCGGAACGTGC
GTGGTGAGGACAGCGGCCGCAAGCCCGAGCGGCGGCGCCTGAAGACCACG
CGTCTGAAGGAGTACACCATCAAGTCGCACTCCAGCTTGCCGCCCAACAA
CAGCTACGCCGACTTCGAGCGCTTCTCCAAGGTGCTGGAGGAGGCGGCGG
CCGCCGAGGAGGGCCTGCGCGAGCTGCAGCGCAGCCGGCGGCTCTGCCAC
GAGGACGTGGAGGCGCTGGCCGCCATCTACGAGGAGAAGGAGGCCTGGTA
CCGCGAGGAGAGCGACAGCCTGGGCCAGGACCTGCGGAGGCTACGGCAGG
AGCTGCTCAAGACCGAGGCGCTCAAGCGGCAGGCGCAGGAGGAGGCCAAG
GGCGCGCTGCTGGGGACCAGCGGCCTCAAGCGCCGCTTCAGCCGCCTGGA
GAACCGCTACGAGGCGCTGGCCAAGCAAGTAGCCTCCGAGATGCGCTTCG
TGCAGGACCTCGTGCGCGCCCTGGAGCAGGAGAAGCTGCAGGGCGTGGAG
TGCGGGCTGCGCTAG
>dapk3_chimpanzee gi|114674686:120-1484 PREDICTED: Pan troglodytes death-associated protein kinase 3 (DAPK3), mRNA, 1365 bases, 40B4791E checksum.
ATGTCCACGTTCAGGCAGGAGGACGTGGAGGACCATTATGAGATGGGGGA
GGAGCTGGGCAGCGGCCAGTTTGCCATCGTGCGGAAGTGCCGGCAGAAGG
GCACGGGCAAGGAGTACGCAGCCAAGTTCATCAAGAAGCGCCGCCTGTCA
TCCAGCCGGCGTGGGGTGAGCCGGGAGGAGATCGAGCGGGAGGTGAACAT
CCTGCGGGAGATCCGGCACCCCAACATCATCACCCTGCACGACATCTTCG
AGAACAAGACGGACGTGGTCCTCATCCTGGAGCTGGTCTCTGGCGGGGAG
CTCTTTGACTTCCTGGCGGAGAAGGAGTCGCTGACGGAGGACGAGGCCAC
CCAGTTCCTCAAGCAGATCCTGGACGGCGTTCACTACCTGCATTCTAAGC
GCATCGCACACTTTGACCTGAAGCCGGAAAACATCATGCTGCTGGACAAG
AACGTGCCCAACCCACGAATCAAGCTCATCGACTTCGGCATCGCGCACAA
GATCGAGGCGGGGAACGAGTTCAAGAACATCTTTGGCACCCCGGAGTTTG
TGGCCCCAGAGATTGTGAACTATGAGCCGCTGGGCCTGGAGGCGGACATG
TGGAGCATCGGTGTCATCACCTACATCCTCCTGAGCGGTGCATCCCCGTT
CCTGGGCGAGACCAAGCAGGAGACGCTCACCAACATCTCAGCCGTGAACT
ACGACTTCGACGAGGAGTACTTCAGCAACACCAGCGAGCTGGCCAAGGAC
TTCATTCGCCGGCTGCTCGTCAAAGATCCCAAACGGAGAATGACCATTGC

CCAGAGCCTGGAACATTCCTGGATTAAGGCGATCCGGCGGCGGAACGTGC
GTGGTGAGGACAGCGGCCGCAAGCCCGAGCGGCGGCGCCTGAAGACCACG
CGTCTGAAGGAGTACACCATCAAGTCGCACTCCAGCTTGCCGCCCAACAA
CAGCTACGCCGACTTCGAGCGCTTCTCCAAGGTGCTGGAGGAGGCGGCGG
CCGCCGAGGAGGGCCTGCGCGAGCTGCAGCGCAGCCGGCGGCTCTGCCAC
GAGGACGTGGAGGCGCTGGCCGCCATCTACGAGGAGAAGGAGGCCTGGTA
CCGCGAGGAGAGCGACAGCCTGGGCCAGGACCTGCGGAGGCTACGGCAGG
AGCTGCTCAAGACCGAGGCGCTCAAGCGGCAGGCGCAGGAGGAGGCCAAG
GGCGCGCTGCTGGGGACCAGCGGCCTCAAGCGCCGCTTCAGCCGCCTGGA
GAACCGCTACGAGGCGCTGGCCAAGCAAGTAGCCTCCGAGATGCGCTTCG
TGCAGGACCTCGTGCGCGCCCTGGAGCAGGAGAAGCTGCAGGGCGTGGAG
TGCGGGCTGCGCTAG
>dapk3_macaque gi|109122940:235-1599 PREDICTED: Macaca mulatta death-associated protein kinase 3, transcript variant 2 (DAPK3), mRNA, 1365 bases, E9AF3C3A checksum.
ATGTCCACGTTCAGGCAGGAGGACGTGGAGGACCATTATGAGATGGGGGA
GGAGCTGGGCAGCGGCCAGTTTGCGATCGTGCGGAAGTGCCGGCAGAAGG
GAACGGGCAAGGAGTACGCGGCCAAGTTCATCAAGAAGCGCCGCCTGTCA
TCCAGCCGGCGCGGGGTGAGCCGGGAGGAGATCGAGCGGGAGGTGAACAT
CCTGCGGGAGATCCGGCACCCCAACATCATCACCCTGCACGACATCTTCG
AGAACAAGACGGACGTAGTCCTCATCCTGGAGCTGGTCTCTGGCGGGGAG
CTCTTCGACTTCCTGGCGGAGAAGGAGTCGCTCACGGAGGACGAGGCCAC
TCAGTTCCTCAAGCAGATCCTGGACGGCGTCCACTACCTGCACTCCAAGC
GCATTGCGCACTTTGACCTGAAGCCGGAAAACATCATGCTGCTGGACAAG
AACGTGCCCAACCCACGCATCAAGCTCATCGACTTCGGCATCGCGCACAA
AATCGAGGCGGGGAATGAGTTCAAGAACATCTTCGGCACCCCAGAGTTTG
TGGCCCCAGAGATTGTGAACTATGAGCCGCTGGGCCTGGAGGCGGACATG
TGGAGCATCGGCGTCATCACCTACATCCTCCTGAGCGGTGCATCCCCGTT
CCTGGGCGAGACCAAGCAGGAGACGCTCACCAACATCTCAGCCGTGAACT
ATGACTTCGACGAGGAGTACTTCAGCAACACCAGTGAGCTGGCCAAGGAC
TTCATTCGCCGGCTGCTCGTCAAAGATCCCAAGCAGAGAATGACCATTGC
CCAGAGCCTGGAACATTCCTGGATTAAGGCCATCCGGCGGCGGAACGTGC
GTGGCGAGGACAGCGGCCGCAAGCCCGAACGGCGGCGCCTGAAGACCACG
CGTCTGAAGGAGTACACCATCAAGTCGCACTCCAGCATGCCGCCCAACAA
CAGCTACGCCAACTTCGAGCGCTTCTCCAAGGTGCTGGAGGAGGTGGCGG
CGGCCGAGGAGGGCCTGCGTGAGCTGGAGCGAAGCCGGCGGCTCTGCCAT
GAGGACGTGGAGGCGCTGGCCGCCATCTACGAGGAGAAGGAGGCCTGGTA
CCGCGAGGAGAGCGACAGCCTGGGCCAGGACCTGCGGCGGCTGCGGCAGG
AGCTGCTCAAGACCGAGGCACTCAAGCGGCAGGCGCAGGAGGAGGCCAAG
GGCGCGCTGCTGGGGACCAGCGGCCTCAAGCGCCGCTTCAGCCGCCTAGA
GAACCGCTACGAGGCGCTGGCCAAGCAGGTGGCCTCCGAGATGCGCTTCG
TACAGGACCTGGTGCGCGCCCTGGAGCAGGAGAAGCTGCAGGGCGTGGAG
TGCGGGCTGCGCTAG
>dapk3_Tupaia 107812812:(1620-1260+1187-1058+844+796+201+175)+108226212:(2038-1886+1173-1128+640-104) Tupaia belangeri (northern tree shrew) Death-associated protein kinase 3 (DAP kinase 3; DAP-like kinase; Dlk; ZIP-kinase); exons from two non-contigous genomic contigs; partial missing N-end, 1303 bases, B19CDD48 checksum.

Fig. S3

CGGCCAGTTCGCCATCGTCCGCAAGTGCCGGCAGAAGGGCACGGGGAAGG
AGTTCGCCGCCAAGTTCATCAAGAAGCGGCGTCTGTCGTCCAGCCGGCGG
GGCGTGAGCCGGGAGGAGATCGAGCGGGAGGTGGACATCCTGCGGGAGAT
CCGGCACCCCAACATCATCGCGCTGCACGACATCTTCGAGAACCGGACGG
ACGTGGTGCTGATCCTGGAGCTGGTGTCGGGCGGCGAGCTCTTCGACTTC
CTGGCCGAGAAGGAGTCGCTGACCGAGGACGAGGCCACCCAGTTCCTGAA
GCAGATCCTGGACGGCGTGCACTACCTGCACTCCAAGCGCATCGCCCACT
TCGACCTCAAGCCAGAGAACATCATGCTACTGGACAAGGACGTGCCCAAC
CCGCGCATCAAGCTCATCGACTTCGGCATCGCCCACAAGATCGAGGCGGG
GAACGAGTTCAAGAACATCTTCGGCACCCCGGAGTTCGTGGCTCCTGAGA
TCGTCAACTACGAGCCCCTGGGCCTGGAGGCCGACATGTGGAGCATCGGC
GTCATCACCTACATCCTCCTGAGCGGCGCGTCCCCGTTCCTGGGCGAGAC
CAAGCAGGAGACCCTGACCAACATCTCGGCGGTGAACTACGACTTCGATG
AGGAGTACTTCGGCAACACCAGCGAGCTGGCCAAGGACTTCATCCGCCGC
CTGCTGGTCAAAGACCCCAAGAGGAGGATGACCATCGCCCAGAGCCTGGA
GCACTCCTGGATCAAGGCGATCCGGCGGCGGAACGTGCGCAGCGAGGACA
GCGGCCGGAAGCCGGAGCGGCGGCGCCTGAAGACGGCGCGGCTGAAGGAG
TACACCATCAAGTCGCACTCCAGCATGCCGCCCAACAACACCTACGTCAA
CTTCGAGCGCTTCTCCAAGGTGCTGGAGGAGGTGGCGGCGGCCGAGGAGG
GCCTGCGCGAGCTGGAGCGCAGCCGGCGGCTGTGCCACGAGGACGTGGCG
GCGCTGACGGCCATCTACGAGGAGAAGGAGGCCTGGTACAGGGAGGAGCA
CGAGAGCATCGGCCAGGACCTGCGGCGGCTGCGGCAGGAGCTGCACAAGA
CCGAGGCGCTCAAGCGCCAGGCGCAGGAGGAGGCCAAGGGCGCCCTGCTG
GGGGCCGGCGGGCTGCGGCGCCGCCTCAGCCGCCTGGAGAACCGCTACGA
GGCGCTGGCCAAGCAGGTGGCCTCCGAGATGCGCTTCGTGCAGGACCTGG
TGCGGGCCATGGAGCAGGAGAAGCTGCAGGCAGTGGGGTGTGGCCTCCGC
TAG
>DAPK3_RAT gi|3250894:114-1460 Rattus norvegicus mRNA for DAP kinase 3 (DLK, ZIPK), 1347 bases, 8F64DA2D checksum.
ATGTCCACGTTCAGGCAGGAGGACGTTGAGGACCATTATGAGATGGGAGA
GGAGCTTGGCAGCGGCCAGTTCGCCATCGTGCGCAAGTGCCAGCAGAAGG
GCACCGGCATGGAGTACGCGGCCAAGTTCATAAAGAAGCGGCGCCTGCCG
TCCAGCCGGCGCGGTGTGAGCCGTGAGGAGATCGAGCGCGAGGTGAGCAT
CCTGCGCGAGATCCGCCACCCCAACATCATCACGCTGCACGATGTGTTCG
AGAACAAGACAGATGTGGTGCTGATCTTGGAGCTGGTGTCCGGCGGCGAA
CTTTTCGACTTTCTGGCTGAGAAGGAGTCACTGACAGAGGATGAGGCCAC
GCAGTTCCTCAAGCAGATCCTGGACGGTGTCCACTACCTGCACTCCAAGC
GCATCGCGCACTTTGACCTGAAGCCGGAGAACATCATGTTGCTGGACAAG
CATGCAGCCAGCCCACGCATTAAGCTCATCGACTTTGGCATCGCGCACAG
GATCGAGGCCGGTAGCGAGTTCAAGAACATCTTTGGCACGCCAGAGTTCG
TCGCCCCTGAGATTGTAAACTATGAACCACTTGGCTTGGAAGCTGATATG
TGGAGCATCGGCGTCATCACCTACATCCTCCTGAGCGGGGCTTCCCCATT
CCTGGGGGAGACCAAGCAGGAAACGCTGACAAACATCTCGGCTGTGAACT
ATGACTTTGATGAAGAGTACTTCAGCAGCACCAGTGAACTGGCCAAGGAC
TTCATCCGAAGGCTGCTGGTCAAAGATCCCAAGAGGAGGATGACCATCGC
ACAGAGCCTGGAGCATTCCTGGATCAAGGTTCGCCGGCGTGAGGACGGCG
CCCGGAAGCCAGAGCGACGCCGACTGCGCGCGGCGCGCCTGCGCGAGTAC
AGTCTCAAGTCGCACTCAAGCATGCCGCGAAACACGAGCTACGCCAGCTT

Fig. S3

CGAGCGCTTCTCGCGCGTGCTGGAGGACGTGGCGGCGGCGGAGCAGGGGC
TGCGCGAGCTGCAGCGGGGCCGGCGGCAGTGCCGGGAGCGCGTGTGCGCG
CTGCGCGTGGCTGCGGAACAGCGGGAGGCGCGCTGCCGCGACGGGAGCGC
CGGGCTAGGCCGCGACCTGCGACGCCTGCGCACGGAGCTGGGTCGCACCG
AGGCTCTGCGCACGCGCGCGCAGGAGGAGGCACGGGCAGCGCTCTTGGGC
GCTGGTGGCCTGAAACGTCGCCTGTGTCGCCTTGAGAACCGCTACGACGC
GCTAGCCGCACAGGTGGCTGCTGAGGTGCAGTTCGTGCGCGATCTGGTAC
GCGCGCTGGAGCAGGAGCGGCTGCAGGCCGAGTGCGGCGTGCGCTAG
>DAPK3_MOUSE gi|2911153:10-1356 Mus musculus mRNA for ZIP-kinase, complete cds, 1347 bases, 9466C0F1 checksum.
ATGTCCACATTCAGGCAAGAGGATGTTGAGGACCATTATGAGATGGGAGA
GGAGCTTGGCAGTGGCCAATTTGCCATCGTGCGCAAGTGCCAGCAGAAGG
GCACGGGCATGGAGTATGCAGCCAAGTTCATCAAGAAGCGGCGCCTGCCA
TCCAGCCGGCGCGGTGTGAGCCGGGAGGAGATCGAACGCGAGGTGAGCAT
CCTGCGCGAGATCCGCCACCCCAACATCATAACACTGCATGACGTGTTCG
AGAACAAGACAGATGTGGTGCTGATCCTGGAGCTGGTGTCCGGTGGCGAG
CTTTTCGACTTCCTGGCCGAGAAGGAGTCATTGACGGAGGATGAGGCCAC
GCAGTTCCTCAAACAAATCCTAGACGGTGTCCACTACCTGCACTCCAAGC
GCATCGCACACTTTGACCTGAAGCCCGAGAACATCATGTTGCTGGACAAG
CACGCAGCCAGCCCCCGCATTAAGCTCATCGACTTTGGCATCGCGCACAG
GATCGAGGCTGGCAGCGAGTTCAAGAACATCTTTGGCACACCCGAGTTTG
TCGCCCCCGAGATCGTGAACTATGAGCCACTTGGCTTGGAGGCTGACATG
TGGAGCATTGGCGTCATCACCTACATCCTCCTGAGCGGAGCGTCCCCATT
CCTGGGCGAGACCAAGCAGGAGACGCTGACGAACATCTCAGCAGTGAACT
ATGACTTTGATGAGGAATACTTCAGCAGCACCAGCGAGCTGGCCAAGGAC
TTCATCCGCAGGCTGCTGGTCAAAGACCCCAAGAGGAGGATGACCATCGC
ACAGAGCCTGGAGCATTCCTGGATCAAGGTGCGCAGGCGCGAGGACGGCG
CCCGGAAGCCAGAGCGACGGCGGCTGCGCGCCGCGCGCCTGCGCGAGTAC
AGCCTCAAGTCCCACTCGAGCATGCCGCGCAACACGAGCTACGCCAGCTT
CGAGCGCTTCTCACGCGTGCTGGAGGACGTGGCGGCGGCAGAGCAGGGGC
TGCGCGAGCTGCAGCGAGGCAGGCGCCAGTGCCGGGAGCGCGTGTGTGCG
CTGCGCGCGGCCGCCGAGCAGCGGGAGGCGCGCTGCCGCGACGGGAGCGC
AGGGCTAGGGCGCGACCTGCGACGCCTGCGCACGGAGCTGGGGCGCACCG
AGGCTCTGCGCACGCGCGCGCAGGAGGAGGCGCGGGCGGCGCTGTTGGGT
GCCGGGGGCCTGAAGCGTCGCCTGTGTCGCCTGGAGAACCGTTACGACGC
GCTAGCCGCTCAGGTGGCCGCTGAGGTGCAATTCGTGCGCGACCTGGTGC
GTGCGCTGGAGCAGGAACGGCTGCAGGCTGAGTGCGGCGTGCGCTAG
>dapk3_cavia assembled_TDB-reads:((812701167+816150362):())+((1587132918+1593835765+760990457+1586592910+812549142+1588856408+816143923+1589066801+1594277302+1603724064+1632773638+1643288589+812697591+1617911338+781611623):(2348-1984+1892-1763+1671-1623+1549-1523+922-769))+((1583699030+810921921):(914-958+1033-1569))+EST_91731898:1-598+EST_148201962:286-384 Cavia porcellus (guinea pig) Death-associated protein kinase 3 (DAP kinase 3; DAP-like kinase; Dlk; ZIP-kinase); partial missing N-end, 1347 bases, 6D922A14 checksum.
GAGGACGTGGAGGACCACTATGAGATGGGGGAGGAGCTGGGCAGCGGCCA
GTTTGCCATCGTGCGCAAGTGCCGTCAGAAGGGCACGGGCAGGGAGTACG
CGGCCAAGTTCATCAAGAAGCGGCGCCTGTCGTCCAGCCGGCGCGGGGTG

Fig. S3

AGCCGCGAGGAGATCGAGCGCGAGGTGAGCATCCTGCGGGAGATCCGGCA
CCCCAACATCATCACACTGCACGATGTCTTCGAGAACAAGACGGACGTGG
TCCTCATCCTGGAGCTGGTGTCCGGCGGCGAGCTCTTTGACTTCCTGGCC
GAGAAGGAGTCGCTGACCGAGGACGAGGCCACGCAGTTCCTCAAGCAGAT
CCTGGACGGCGTCCACTACCTGCACTCCAAGCGCATTGCCCACTTCGACC
TCAAGCCCGAGAACATCATGCTGCTGGACAAGAGCGTGCCCAGCCCGCAC
ATCAAGCTCATCGACTTCGGCATCGCCCACAAGATTGAGGCCGGGAATGA
GTTCAAGAACATCTTCGGCACCCCCGAGTTCGTGGCCCCCGAGATCGTCA
ACTACGAGCCGCTGGGCCTGGAGGCTGACATGTGGAGCATTGGTGTCATC
ACCTATATCCTGCTCAGCGGAGCATCCCCCTTCCTGGGGGAGACCAAGCA
GGAGACGCTGACCAACATCTCTGCAGTGAATTACGACTTTGACGAGGAAT
ATTTCAGCAACACGAGTGAGCTGGCCAAGGACTTCATCCGCCGCCTGCTG
GTCAAGGACCCCAAGAGGAGGATGACCATCGCGCAGAGCCTGGAGCACTC
CTGGATCAAGGCCATCCGGAGGCGCAACGTGCGCGGCGAGGACGGCGGGC
GCAGGCCGGAGCGGCGGCGGCTGCGCGCGGCGCGCCTGAAGGAGTACACC
ATCAAGTCGCACTCCAGCATGCCGCCCAACAACAGCTACGCCAGCTTCGA
GCGCTTCTCGCGCGTGCTGGAGGAGGTGGCGGCGGCCGAGGAGGGGCTGC
GCGAGCTGCAGCGCGGCCGGCGGCTGTGCCGCGAGGACGTGGAGGCGCTG
GCCGCCATCTACGAGGAGAAGGAGGCCTGGTACACGGAGGAGCGCGACAG
CATCGGCCGCGACCTGCGGCGCCTGCGGCAGGAGCTGCACAAGACGGAGG
CGCTGCAGCGGCAGGCGCAGGAGGAGGCCAAGGGCGCGCTGCTGGGCGCC
AGCGGGCTCAAGCGCCGCTTCAGCCGCCTGGAGAACCGCTACGAGGCGCT
GGCCAAGCAGGTGGCGTCCGAGATGCGCTTCGTGCAGGAGCTGGTGCGCG
CGCTGGAGCAGGAGCGGCTGCAGGGCGGGGAGTGCGGCCTGCGCTAG
>dapk3_ Kangaroo rat assembled_TDB-
reads:((1527350676+1542302641+1556273330+1541211393+1578971935+1596066105+156291
2636+1552706462):(1164-
1523))+((1569549916+1566271599+1585076393+1585061768+1542035913+1556201828+15412
00922+1586154871+1585648556+1535177412):(26-10+317-365+832-858+1502-1655+2363-
2407))+(1569544450:112-22)+(1587085697:29-324) Dipodomys ordii (Ord's kangaroo
rat; a rodent) Death-associated protein kinase 3 (DAP kinase 3; DAP-like kinase;
Dlk; ZIP-kinase); partial missing N and C ends, an internal exon, and part of an
exon (shown as Ns), 1160 bases, 676C6F68 checksum.
GGCCAGTTTGCCATCGTGCGCAAGTGCCGGCAGAAGGGCACGGGCAAGGA
GTACGCCGCCAAGTTCATCAAGAAGCGCCGCCTGTCATCCAGCCGGCGGG
GCGTGAGCCGTGAGGAGATCGAGCGCGAGGTGAGCATCCTGCGGGAGATC
CGGCACCCCAACATCATCACGCTGCACGACGTCTTTGAGAACAAGACCGA
CGTGGTGCTCATCCTGGAGCTCGTGTCCGGCGGGGAGCTCTTCGACTTCC
TGGCCGAGAAGGAATCGCTGACGGAGGATGAGGCCACGCAGTTCCTCAAG
CAGATCCTGGACGGCGTCCACTACCTGCACTCCAAGCGCATTGCCCACTT
TGACCTCAAGNNNNNNNNNNNNNNNNNNNNNNNNNNNNNNNNNNNNNNNN
NNNNNNNNNNNNNNATCGACTTCGGCATCGCCCACAAGATCGAGGCTGGC
AACGAGTTCAAGAATATCTTTGGCACGCCTGAATTTGTGGCTCCTGAGAT
TGTTAACTACGAGCCCCTGGGCCTGGAGGCAGACATGTGGAGCATCGGCG
TCATCACCTACATCCTCCTCAGCGGCGCGTCCCCGTTCCTGGGGGAGACC
AAGCAGGAGACCCTGACCAACATCTCTGCTGTGAACTACGACTTTGATGA
GGAGTACTTCAGCAGCACCAGCGAGCTGGCCAAGGACTTCATCCGCCGCC
TCCTGGTCAAAGACCCCAAGAGAAGGATGACCATCGCCCAGAGCCTGGAA

CATTCCTGGATCAAGGCCATCCGGCGGCGCGGTCCGCAGCGAGGACGG
CGGGAGGCGCGCGGAGCGGCGGCGCCTGCGCACCACGCGGCTGAAGGAGT
ACACCNNNNNNNNNCACTCGAGCATGCCGCCCAACAACTCGTACGCCAAC
TTCGAGCGCTTCTCCAAGGTGCTGGAGGAGGTGGCGGCGGCCGAGGAGGG
GCTGCGCGAGCTGGAGCGCGGCCGGCGGCTGTGCCGCGAGGACGTGGAGG
CGCTGGCGGCCATCTACGAGGAGAAGGAGGCCTGGTACCGCGAGGAGAGC
GCCGGCCTGGGCCAGGACGTGCGGCGCCTGCGCCAGGAGCTGCACCGCGC
CGATGCGCTGCGGCGCCAGGCGCACGAGGAGGTCCGGGCGGCGCTGCTGG
GCTCTGGAAG
>dapk3_Dog gi|73987436:1-1365 PREDICTED: Canis familiaris similar to Death-associated protein kinase 3 (DAP kinase 3) (DAP-like kinase) (Dlk) (ZIP-kinase) (LOC476745), mRNA, 1365 bases, F40F6429 checksum.
ATGTCCACATTCAGGCAGGAAGACGTGGAAGACTACTATGAGATGGGGGA
GGAGCTGGGCAGCGGCCAGTTTGCGATTGTGCGGAAATGCCGGCAGAAGG
GGACTGGGAAGGAGTATGCGGCCAAGTTCATCAAGAAGCGCCGCCTGTCG
TCCAGCCGGCGGGGAGTAAGCCGGGAGGAGATCGAGCGGGAGGTGAACAT
CCTACGGGAGATCCGGCACCCCAACATCATCACGCTGCACGACATCTTTG
AGAACAAGACTGACGTAGTGCTCATCCTGGAGCTGGTCTCAGGCGGGGAG
CTCTTCGACTTCCTGGCAGAGAAGGAGTCACTGACAGAGGACGAGGCCAC
CCAGTTCCTCAAGCAGATCCTGGATGGTGTCCACTACCTGCACTCCAAGC
GCATTGCCCACTTTGACCTCAAGCCAGAAAACATCATGCTGCTAGACAAG
AACGTGCCCAACCCACGGATCAAGCTCATCGACTTTGGCATCGCCCACAA
GATCGAAGCGGGGAATGAGTTCAAGAACATTTTCGGCACCCCTGAATTTG
TGGCTCCAGAGATTGTCAACTATGAACCCCTGGGTCTGGAAGCAGACATG
TGGAGCATTGGAGTCATCACCTATATCCTCCTGAGCGGCGCCTCCCCATT
CCTGGGTGAGACCAAGCAGGAGACCCTGACCAACATCTCGGCTGTGAACT
ACGACTTTGACGAGGAGTATTTCAGCAACACCAGCGAGCTGGCCAAGGAC
TTCATCCGCCGCCTGCTTGTCAAGGACCCCAAGAGAAGAATGACCATCGC
CCAGAGCCTGGAGCATTCCTGGATCAAGGCGATCCGGCGGCGGAACGTGC
GGCGTGAAGACAGTGGCCGGAAGCCCGAGCGGCGGCGCCTGAAGACGGCA
CGCCTCAAGGAGTACACCATCAAGTCCCACTCGAGCATGCCCCCCAACAA
CACCTATGTCAACTTTGAGCGCTTCTCCAAGGTGCTAGAGGAGGTGGCGG
CGGCAGAGGAGGGTCTGCGGGGGCTGGAGCACAGCCGGCGCCTCTTCCAC
GAGGACATCGAGGCGCTGACGGCCATCTACGAGGAGAAGGAGGCCTGGTA
CAGGGAGGAGAACGAGAGCATCGGCCAGGACCTGCGGCGGCTGCGCCAGG
AGCTGCACAAGACGGAGGCGTTGCAGCGGCAGGCCCAGGAGGAGGCCAAG
GGCGCCCTGCTGGGGGCCAGTGGGCTCAAGCGGCGCTTCAGCCGCCTCGA
GAACCGCTACGAGGCCCTGGCAAAGCAGGTGGCCTCCGAGATGCGCTTCG
TGCAGGATCTGGTGCGCGCCATGGAGCAGGAGAAGCTGCAGGGGGTGGAG
TGTGGCCTACGCTAG
>dapk3_Cow gi|119894932:194-1558 PREDICTED: Bos taurus similar to ZIP-kinase (LOC525506), mRNA, 1365 bases, 41A61006 checksum.
ATGTCAACATTCAGGCAGGAGGATGTGGAAGACCACTATGAGATGGGAGA
GGAGCTGGGAAGCGGACAGTTTGCAATTGTGCGGAAGTGCCGGCAGAAGG
GTACTGGGAAGGAGTATGCAGCCAAGTTCATCAAGAAGCGCCGCCTCTCG
TCCAGCCGCCGGGGAGTGAGCCGAGAGGAGATCGAGCGGGAGGTGAACAT
CCTGCGGGAGATCCGGCACCCCAACATCATCACACTGCATGACATCTTTG
AGAACAGGACGGACGTGGTGCTTATCCTCGAGCTGGTCTCAGGTGGTGAG

CTCTTCGACTTCCTGGCCGAGAAGGAGTCACTGACCGAGGACGAGGCCAC
CCAGTTCCTCAAGCAGATCCTGGATGGTGTCCACTATCTGCACTCCAAGC
GCATCGCCCACTTCGACCTCAAGCCAGAAAACATCATGCTCCTGGACAAG
AATGTGCCCAACCCACGGATCAAGCTCATTGACTTTGGCATCGCCCACAA
GATCGAGGCAGGGAACGAGTTCAAGAACATCTTTGGGACCCCGGAGTTCG
TGGCTCCTGAAATTGTCAACTATGAGCCCCTGGGTCTGGAGGCAGACATG
TGGAGCATTGGCGTTATCACCTACATCCTGCTGAGTGGCGCGTCCCCGTT
CCTGGGTGAAACCAAGCAGGAGACCCTGACCAACATCTCGGCTGTGAATT
ACGACTTTGACGAGGAGTACTTCAGCAACACCAGCGAGCTGGCGAAGGAC
TTCATCCGCCGTCTGCTTGTCAAAGACCCCAAGAGGAGAATGACCATCGC
CCAGAGCCTGGAGCACTCCTGGATCAAGGCCATCCGGCGGAGGAATGTGC
GGCGGGAGGACAGCGGCCGGAAGCCAGAGCGGCGGCGCCTGAAGACGGCA
CGCCTCAAGGAGTACACCATCAAGTCGCACTCAAGCATGCCCCCCAACAA
TACCTATATCAACTTCGAGCGCTTCTCCAAAGTGCTGGAGGAGGTGGCGG
CGGCCGAGGAGGGCCTGCGCGGGCTGGAGCACAGCCGACGCCTGTTCCAT
GAGGACCTTGAGGCGCTGACGGCCATCTACGAGGAGAAGGAGGCCTGGTA
CCGTGAGGAGAACGAGAGCCTAGGCCAGGACCTGCGGCGGCTGCGCCAGG
AGCTGCACAAGACGGAGGCGCTGCAGCGGCAGGCACAGGAGGAGGCCAAG
GGGGCCCTGCTCGGGGCCAGTGGGCTCAAGCGCCGCTTTAGCCGCCTGGA
GAACCGCTACGAGGCGCTGGCCAAGCAGGTGGCCTCCGAGATGCGGTTCG
TGCAGGATCTGGTACGCGCCATGGAGCAGGAGAAGCTGCAAGAGGGGGAG
TGCAGCCTCCGCTAG
>dapk3_Bat 105860175:(6-134+762-810+4383-4409+5004-5156+5920-5965+6421-6957)+assembled_TDB-reads((976759098+964301557+981846460):(456-96)) Myotis lucifugus (little brown bat) Death-associated protein kinase 3 (DAP kinase 3; DAP-like kinase; Dlk; ZIP-kinase); partial missing N-part, two contigs joined by a joint mate pair, 1303 bases, A3F80AC6 checksum.
CGGCCAGTTTGCCATTGTGCGGAAATGCCGGCAGAAGGGCACTGGGAAGG
AGTATGCAGCCAAGTTCATCAAGAAACGCCGCCTGTCGTCCAGCCGGCGG
GGGGTCAGCCGGGAGGAGATCGAGCGGGAGGTGAACATCCTGCGGGAGAT
TCGGCACCCCAATATCATCACGCTACATGACATCTTTGAGAATAAGACCG
ATGTGGTGCTCATCCTGGAACTGGTTTCTGGTGGGGAGCTCTTTGATTTC
TTGGCTGAGAAGGAGTCGCTGACAGAGGACGAGGCCACTCAGTTCCTTAA
GCAGATCCTGGATGGCGTCCACTACCTACACTCCAAGCGCATCGCCCACT
TTGACCTCAAGCCCGAAAACATCATGCTCCTAGACNAGAACGTGCCCAAC
CCACGCATCAAGCTTATTGACTTTGGCATCGCTCACAAGATTGAAGCCGG
GAACGAGTTCAAGAACATCTTTGGCACCCCAGAATTTGTTGCTCCTGAGA
TTGTCAACTATGAACCCCTGGGTCTGGAGGCGGACATGTGGAGCATTGGT
GTCATCACCTATATCCTTTTGAGTGGCGCATCCCCATTCCTGGGTGAGAC
CAAGCAGGAGACCCTGACCAATATCTCAGCCGTGAACTATGACTTCGATG
AGGAGTACTTTAGCAACACCAGCGAGCTGGCCAAGGACTTCATCCGCCGG
CTGCTTGTCAAGGACCCCAAGAGGAGAATGACCATCTCCCAGAGCCTGGA
GCATTCCTGGATCAAGGCGATCAGGCGGCGGAATGTGCGGAGGGAAGACA
GTGGCCGGAAACCGGAGCGTCGGCGCCTGAAGACAGCCCGCCTCAAGGAG
TACACTATCAAGTCCCATTCGAGCATGCCCCCCAACAATACCTACATCAA
CTTCGAGCGCTTCTCCAAGGTACTGGAGGAGGTGGCGGCGGCCGAGGAGG
GCCTGCGTGGGCTGGAACACAGCCGGCGGCTCTTCCATGAGGACATCGAG
GCCCTGACAGCCATCTATGAAGAGAAGGAGGCCTGGTACAGGGAGGAGAA

CGAGAGCATTGGCCAGGACCTGCGACGGCTGCGGCAAGAGCTGCACAAGA
CCGAGGCCCTCCAGCGGCAGGCCCAGGAGGAGGCCAAGGGTGCCCTGCTA
GGGGCCAGCGGGCTCAAGCGCCGCTTCAGCCGCCTTGAGAACCGCTACGA
GGCTCTGGCCAAGCAGGTGGCCTCCGAGATGCGGTTTGTGCAGGATCTTG
TACGCGCCATGGAGCAGGAGAAGCAACAGGAGGTGGAATGTGGCCTCCGC
TAG
>dapk3_Shrew 80402909:(1-324,1034-1163,1349-1397,1738-1764,2080-2232;+FS_corrections)+80402905:154-690+assembled_TDB_reads:((838222606+892417228):(1033-713))+((873967520+875830371+894062723+845045749+848969792):(478-520+770-1307)) Sorex araneus (European shrew) Death-associated protein kinase 3 (DAP kinase 3; DAP-like kinase; Dlk; ZIP-kinase); concatatnation of CDS from two contigs; partial missing N-part, 1263 bases, 6F8AB504 checksum.
GGCACGGGCAAGGAATACGCGNNNNNNNNNNNNNNNAAGCGGCGCCTGTC
ATCCAGCCGGCGCGGGTGNAGCCGGGAGGAGATCGAGCGGGAGGTGGACA
TCCTGCGGGAGATCCGGCACCCCAACATCATCACGCTGCACGACGTGTTC
GAGAACAAGACGGACGTGGTGCTCATCCTGGAGCTGGTGTCCGGCGGCGA
GCTCTTCGACTTCCTGGCCGAGAAGGAGTCGCTGACCGAGGACGAGGCCA
CGCAGTTCCTCAAGCAGATCCTGGACGGCGTCCACTATCTGCACTCGAAG
CGCATCGCCCACTTTGACCTCAAACCGGAGAATATCATGCTTCTGGACAA
GAACGTCGCCAGTCCCCGGATCAAGCTCATTGACTTCGGCATCGCCCACA
AGATCGAAGCCGGGAACGAGTTCAAGAACATCTTCGGCACCCCGGAGTTT
GTGGCTCCTGAGATTGTCAACTACGAGCCCCTGGGCCTGGAGGCTGACAT
GTGGAGCATCGGCGTTATCACCTACATCCTCTTGAGCGGCGCGTCCCCGT
TCCTGGGGGACACCAAGCAGGAGACGCTGACCAACATCTCGGCCGTCAAC
TACGACTTCGATGAGGAGTATTTCAGCAGCACCAGCGAGCTGGCCAAGGA
CTTCATCCGCCGGCTCCTGGTCAAGGACCCCAAGAGAATGACCATTGCCG
AGAGCCTGGAGCACTCCTGGATCAAGGCCATCCGGCGGCGGAACGTGCGG
CGGGAGGACAGCGGCAAGAAGCCAGAGCGGCGGCGCCTGAAGACCGCGCG
CCTCAAGGAGTACACCATCAAGTCGCACTCCAGCATGCCCCCCAACCACA
CCTACATCAACTTCGAGCGCTTCTCCAAGGTGCTGGAGGAGGTGGCGGCG
GCCGAGGAGGGCCTGCGCGGCCTGGAGCACAGCCGGCGCCTGTGCCACGA
GGACGTGGAGGCACTCACGGCCATCTACGAGGAGAAGGAGGCCTGGTACC
GCGAGGAGCAGGAGGGCCTGGGCCAGGACCTGCGGCGGCTGCGCCAGGAG
CTGCACAAGACCGAGACGCTGCGGCGCCAGGCCCAGGAGGAGGCCAAGGG
CGCGCTGCTGGGCGCCAACGGGCTCAAGCGCCGCTTCAGCCGCCTGGAGA
ACCGCTACGAGGCCCTGGCGCAGCAGCTGGCCTCCGAGATGCGCTTCGTG
CAGGACCTGGTGCGCACCATGGAGCAGGAGAAGCTGCAGGGCGGCCAGTG
CGGGCTGCGCTAG
>dapk3_Opossum 84819837:(42776-43312+44038-44083+44551-44703+45397-45423+46553-46601+46993-47122+47716-48076+51934-51995) Monodelphis domestica (opossum) Death-associated protein kinase 3 (DAP kinase 3; DAP-like kinase; Dlk; ZIP-kinase), 1365 bases, B083636 checksum.
ATGTCTACGTTCAGGCAGGAGGATGTGGAAGACCACTATGAGATGGGTGA
GGAGCTGGGCAGTGGTCAGTTTGCAATTGTACGGAAATGTCGTCAGAAGA
GCAATGGTATGGAGTATGCAGCTAAGTTTATCAAGAAGCGACGCTTGTCA
TCCAGCCGTCGCGGGGTGAGCCGGGAGGAGATAGAAAGGGAGGTGGACAT
TCTTCGGGAAATCCAGCACCCTAACATCATTACCCTTCATGACATCTTTG

AGAACAAGACTGATGTAGTATTAATCTTGGAACTGGTTTCTGGGGGTGAG
CTCTTTGATTTCTTGGCTGAAAAGGAGTCCCTAACTGAGGAAGAGGCTAC
CCAGTTCCTTAAGCAGATCTTAGATGGTGTCCATTATCTGCACTCCAAGC
GAATTGCCCATTTTGATTTAAAGCCAGAGAACATCATGCTTCTGGATAAG
AATGTGCCCAACCCTCGAATCAAGCTCATTGATTTTGGTATTGCCCATAA
AATTGAGGCAGGGAATGAGTTCAAGAACATTTTTGGAACCCCAGAGTTTG
TGGCTCCTGAGATTGTGAACTATGAACCATTAGGATTGGAGGCTGATATG
TGGAGCATTGGCGTGATCACATACATTCTTTTGAGTGGAGCATCACCATT
CTTGGGGGAAACCAAGCAGGAGACTCTCACCAACATTTCAGCTGTGAACT
ATGACTTTGATGAAGAGTATTTCAGTAATACCAGCGAGCTGGCCAAGGAC
TTCATCCGACGACTGCTTGTCAAAGATCCTAAGAAAAGGATGACCATTGA
CCAGAGCCTGGAGCATTCATGGATTAAGGTGATTAAGAGGAGGAATGTGC
GGAATGAGGACAGTGGCAAGAAGCCAGAGCGGCGTCGGCTGAAGACAACC
CGCCTCAAGGAGTACACTATCAAGTCTCACTCAAGCATGCCCCCAAACAA
CACATACATTAATTTTGAGCGCTTCTCTAAGGTTCTCGAGGAGGTGGCAG
CAGCTGAAGAGGGCCTTCGGGAGCTGGAACGAAGCAAGAAACTGTTCCGT
GAGGATATTGAAGTCCTAACTTCGATCTATGAGGAGAAGGAAGCCTGGTA
TAAGGAGGAAAATGAGAGCATTGGCCAGGATCTGAGGCTGCTTCGTCAGG
AACTGCACAAGACAGAGGCACTGAAACGGCAGGCCCAAGAAGAGGCCAAG
GGGGCTCTGCTGGGGGCTAATGGGCTGAAACGCCGCTTCAGCCGCCTAGA
GAACCGCTATGAGGCCCTGGTTAAGCAAGTGGCATCAGAGATGAGGTTTG
TTCAGGACCTGGTTTATGCCATGGAGCAGGAGAAAATGCAGGATGGGGAC
TGCAGTGTGCGTTAG
>dapk3_Platypus 91473547:(3474-3834+4007-4136+5210-5258+6263-6289+6613-6765+8023-8077+8407-8934) Ornithorhynchus anatinus (platypus) Death-associated protein kinase 3 (DAP kinase 3; DAP-like kinase; Dlk; ZIP-kinase); partial missing N-end, 1303 bases, 5BC04AE5 checksum.
CGGTCAGTTTGCGATTGTGCGGAAGTGTCGGCAGAAGAGTAATGGGCTGG
AGTACGCGGCCAAGTTCATCAAAAAGCGTCGCCTGTCGTCCAGCCGGCGG
GGGGTGAGCCGGGAAGAGATCGAGCGGGAAGTGGACATCCTGAGGGAGAT
TCAACACCCCAACATCATCACCCTGCACGACATCTTTGAGAACAAGACGG
ACGTGGTGCTGATCCTGGAGCTGGTCTCTGGCGGGGAGCTCTTTGACTTT
CTGGCTGAGAAAGAGTCCCTGACTGAGGAGGAGGCCACTCAGTTTCTGAA
GCAGATCCTGGATGGGGTCCACTACCTGCACTCCAAGCACATTGCCCACT
TTGACCTAAAGCCTGAGAACATCATGCTTCTGGACAAGAATGTGCCCAAT
CCTCGCATCAAGCTGATCGACTTTGGCATTGCCCACAAAATTGAGGCCGG
GAATGAGTTCAAGAACATCTTTGGACCCCAGAGTTCGTGGCTCCTGAGA
TCGTGAACTACGAGCCCCTGGGGCTAGAGGCTGACATGTGGAGCATTGGA
GTGATCACCTACATCCTTTTAAGCGGCGCATCTCCATTCTTGGGTGAAAC
CAAACAAGAGACCCTGACCAACATCTCGGCGGTGAACTATGACTTTGATG
AGGAATATTTCAGTAACACCAGCGAGCTGGCCAAGGACTTCATCCGCCGA
CTACTCGTCAAGGACCCCAAGAAGAGGATGACCATTGCCCAGAGCCTGGA
GCACTCCTGGATTAAGGTGAGGAGAAGGAGGAACGTGCGGAACGAGGACA
GCGGCAAGAAGCCCGAGCGGCGGCGGCTGAAGACCACCCGGCTCAAGGAG
TACACCATCAAGTCCCACTCCAGCATGCCCCCCAACAACACCTACATCAA
CTTCGAGCGCTTCTCCAAGGTGCTGGAGGAAGTGGCAGCGGCCGAGGAGG
GCCTGAGGGAGCTGGAGCACAGTAAGAAGCTCTTCCGGGAGGACATCGAG
GCCCTGACCTCCATCTACGAAGAGAAGGAGGCCTGGTACAAGGAGGAGAA

CGAGAGCATCGGCCAAGACCTGCGGCAGCTCCGCCAGGAGCTCCACAAGA
CGGAGGCCCTGAAGCGGCAGACCCAGGAAGAGGCCAAGGGGGCCCTGCTC
GCCGCCAACGGGCTCAAGCGGGGCTTCGGGCGGCTGGAGAACCGCTTCGA
GGCCCTGGCCAAGCAGGTGGCGTCTGAGATGAGGTTCGTGCAGGACCTGG
TGCGGGCCATGGAGCAGGAGAAGCTGCAGGAGAGCGACTGCAGCATCCGT
TAG
>dapk3_Chicken ((NC_006115.1:1990-1980K):(2069-2130+2706-3066+3912-4041+4262-4310+4412-4441;4641-5162))+(EST-25936497:4-659)+(BBSRC_EST-604156336F1:1-552)+(TDB_read|227294367:(31-86+308-256+458-485+564-711)) Gallus gallus (chicken) Death-associated protein kinase 3 (DAP kinase 3; DAP-like kinase; Dlk; ZIP-kinase); assembly of genomic contig, ESTs and a sequence read, 1374 bases, C471AF85 checksum.
ATGTCCACCTTCCGGCAGGAGAGCGTGGAGGACTTCTACGAGATGGGGGA
AGAGCTGGGCCACGGCCAATTCGCCATCGTGAGAAAATGCCGGGAGAGGA
AAACGGGCGTGGAATACGCGGCCAAATTCATCAAGAAGCGGCGGCTGTCG
TCGAGCCGTCGGGGTGTGAGCAGGGAGGAGATCGAGAGGGAGGTGGACAT
CCTGAGGGAGATCCAACACCCCAACATCATCACACTGCACGACATCTTCG
AGAACAAGACGGACGTGGTGCTGATCCTGGAGCTGGTGTCGGGCGGGGAG
CTCTTCGACTTCCTGGCCGAGAAGGAATCGCTGACGGAGGAGGAGGCCAC
GCAGTTCCTCAAGCAGATCCTGGACGGGGTGCATTACCTGCATTCCAAGC
GCATCGCACACTTTGACTTGAAGCCAGAGAACATCATGCTGCTGGATAAG
AACGTGCCCAACCCTCGCATCAAACTCATTGACTTCGGGATCGCCCACAA
GATTGAAGCTGGGAATGAGTTCAAGAACATATTTGGGACCCCGGAGTTCG
TGGCACCAGAAATCGTGAACTACGAGCCGCTGGGGCTGGAGGCCGACATG
TGGAGCATTGGGGTCATCACTTACATCCTGGAGAGCGGGGCGTCCCCCTT
CCTGGGGGAAACGAAGCAGGAGACCCTCACCAACATCTCCGCCGTCAACT
ACGACTTCGATGAGGAGTACTTCAGCAGCACCAGCGAGTTGGCCAAGGAC
TTCATCCGCCGGCTGCTGGTCAAGGACCCCAAGAAGAGGATGACCATAGC
TCAGAGCCTGGAGCACCCCTGGATCAAGGTGATCAAGAGGAGGAACGTCC
GCAACGAGGACAGCGGGAAGAAGGCGGAGCAGCGGCGGCGGTTGAAGACG
ACGCGGTTGAAGGAATACACCATCAAGTCCCACTCCAGCATGCCGCCCAA
CAACACCTACGTCAACTTCGAGCGCTTCTCCAAAGTGATGGAGGAGGTGG
CGGCGGCGGAGGAGACCCTCCGCGAGATACAGAGGAACAAGAGATCCTTC
CGGGAGGACATCGAGGCCCTGCTGTCCATCTACGAGGAGAAGGAGTCGTG
GTACAAAGAGGAGAATGAGAGCATCGGCCGCGACCTGCGGCAGATCCGGC
AGGAGCTGCTCCGAGCTGAGTCCGCCCGGCAGCGGGCGCAGGAGGAGAGC
AGGAATGTGATGCAGGCGGCCGGGGGGCTGCGCCGGCGCTACCGGAGGTT
GGAGAAGCGCTACGGTGCCCTGGCCGAGCAGGTGGCGTCCGAGGTGAGGT
TCGTGCAGGAGCTGCTTTGGTCCATCGAGAGGGAGAAGCTGCAGAGCGGC
GGAGAGGATGGAAGCATCCGCTGA
>dapk3_Finch assembled_TDB-reads_(1241607171:(781-720+191-68))+((1242613131+1422732028+1404775456+1277318751):(530-890))+((1401310278+1277343563+1253220430+1397767862+1249786453+1290200497+1398826774+1397636686+1423694077):(567-696+839-887+994-1020+1120-1272+1647-1692+1833-2375)) Taeniopygia guttata (zebra finch) Death-associated protein kinase 3 (DAP kinase 3; DAP-like kinase; Dlk; ZIP-kinase), 1371 bases, 711E18F5 checksum.
ATGTCCACTTCCGCCAGGAGAAGCGTGGAGGACTTCTATGAGATGGGCGA
AGAGCTGGGCAGCGGGCAGTTCGCCATCGTCCGCAAGTGCCGGGAGAGGA

Fig. S3

AGACGGGCCTGGAGTACGCGGCCAAGTTCATCAAGAAGCGGCGGCTGTCG
TCCAGCCGCCGGGGCGTGAGCCGCGAGGAGATCGAGCGCGAGGTGGACAT
CCTGCGCGAGATCCAGCACCCCAACATCATCACCCTGCACGACATCTTCG
AGAACAAGACCGACGTGGTGCTCATCCTGGAGCTGGTGTCCGGCGGGGAG
CTCTTCGACTTCCTGGCCGAGAAGGAGTCCCTGACCGAGGAGGAGGCCAC
CCAGTTCCTCAAGCAGATCCTGGACGGCGTGCACTACCTGCACTCCAAGC
GCATCGCCCACTTCGACTTGAAGCCAGAGAACATCATGCTGCTGGACAAG
AACGTGCCAAACCCTCGCATCAAACTCATCGACTTTGGGATCGCCCACAA
GATCGAGGCTGGGAATGAGTTCAAGAATATCTTTGGGACCCCAGAGTTTG
TAGCCCCTGAAATTGTGAACTACGAGCCCCTGGGGCTGGAGGCCGACATG
TGGAGCATCGGGGTCATCACCTACATCCTGCTGAGCGGAGCCTCCCCGTT
CCTGGGGGAGACCAAGCAGGAGACACTGACCAACATCTCTGCTGTCAACT
ACGACTTCGATGAGGAATATTTCAGCAACACCAGCGAGCTGGCCAAGGAC
TTCATCCGCCGCCTGCTCGTCAAGGACCCCAAGAAGCGAATGACCATCGC
CCAGAGCCTGGAGCACCCGTGGATTAAGGTGATCAAGAGGAGGAACGTCC
GCAACGAGGACAGCTGCAAGAAGCCCGAGCGGCGCCGGCTGAAGACCACG
CGCCTGAAGGAGTACACCATCAAATCCCACTCCAGCATGCCCCCCAACAA
CACTTACATCAACTTCGAGCGCTTCTCCAAGGTCATGGAGGAGGTGGCTG
CGGCCGAGGAGAGCCTCCGCGAGCTGGAGAGGAACAAGAAGTCCTTCCAG
GAGGACATCGAGGCGCTGCTGTCCATCTACGAGGAGAAGGAGTCGTGGTA
CAAGGAGGAGAACGAGAGCATCAGCCAGGACCTGCGGCAGATCCGGCAGG
AGCTGCAGAAGACGGAGGCGCTGAAGAAGCAGGCGCAGGAGGAGACCAAG
AGCGTGGTGCAGGCGGCCAACGGGCTCAAGCGGCGCTACCGCAAGCTGGA
GAACCACTACGAGGCCCTGGCCAAGCAGGTGGCCTCTGAGATGAAGTTTG
TGCAGGAGCTGGTGTGGTCCATCGAGCGGGAGAAGCTGCAGAGCAGCGAG
GGCGACGGCAGCATCCGCTAA
>dapk3_xenla gi|66911520:79-1437 Xenopus laevis hypothetical protein MGC114871, mRNA (cDNA clone MGC:114871 IMAGE:5506395), complete cds, 1359 bases, F38A16EF checksum.
ATGAGCGGATTCCGGCAGCAGAATGTCGATGACTTTTATGAAATGGGAGA
AGAATTGGGCAGCGGGCAGTTTGCAATTGTGCGGAAGTGCCGGGAGCGTT
CTACAGGTGTGGAGTATGCAGCTAAATTTATTAAGAAACGCCGATTGTCA
TCTAGCCGCCGAGGTGTGAGCCGAGAAGAAATTGAGAGGGAGGTAGACAT
ACTAAGAGAGATTCAGCACCCCAACATCATCACCTTACATGATGTGTTTG
AGAATCGCACAGATGTGGTCCTGATACTTGAGCTTGTTTCTGGAGGGGAG
CTTTTTGACTTTCTGGCACAAAAGGAAAGTTTAAGTGAGGAAGAGGCAAC
CATGTTCCTGAAGCAGATACTTGATGGTGTTCATTATTTGCACCATAAGA
ATATTGCACATTTTGACCTTAAGCCAGAGAATATCATGCTTTTGGACCAG
AGTTCCCCCAGTCCAAGAATAAAACTCATAGATTTTGGAATTGCTCACAA
GATAGAGGCTGGGAATGAATTCAAAAATATCTTTGGAACTCCTGAATTTG
TAGCTCCAGAAATCGTAAACTATGAGCCTTTGGGACTGGAAGCTGATCTG
TGGAGCATTGGAGTTATCACTTACATTCTTCTCAGTGGAGCATCACCATT
TCTTGGAGACACCAAGCAGGAAACGTTGACTAACATTTCAGGTGTGAATT
ATGATTTTGATGAAGAATATTTCAGCAATACCAGTGAATTAGCTAAAGAT
TTTATTCGCCGGCTGTTGGTGAAAGATCCAAAAAAACGAATGACCATCGA
TCAGAGTTTAGAGCATTCATGGATTAAGGCTATCAAGCGCCGAAATGTCC
GACATGAGGACAGTGGAAAACGCCCTGAACGCCGTCGCCTGAAGACTACA
CGTTTAAAGGAATACACAATAAAATCACATTCTAGCATGCCTCCAAATAA

CACCTATGTGAATTTTGAAAGGTTTAGCCATGTATTAGAGGAGCTGGGAG
CAGCAGAAGATGCACTTCGTGAGCTTCAAAAGAGTCAGGCCACCTCACGC
GAAGACCTGTGTGCCTTGCGCTCTATCTACGAGGAAAAGGAAAGTTGGTA
CCATGAAGAGCAGGAAGGTGTTGGGCGTCAACTTGTAGGGCTCCGGCAAG
AACTGCAGCGCAGAGAGAGTCAGCGTCGGCAGGTACAGGAGGATTTAAGG
GGAGCTATGCTTAGTGCCAATGCTTTGAAGAGAAGGTTTGGTAAATTGGA
AAATAGATATGAGGCCCTGCAGGCACAATTTACTGCAGAAATGAGCTTTG
TGGAAGATTTGTTAAAAACTGTGGAGAGAGGTGAAGATGGATTAAGAGGA
GTGTGCTGA
>dapk3_xentr gi|77622189:45-1403 Xenopus tropicalis finished cDNA, clone TGas051f01, 1359 bases, E50E372C checksum.
ATGAGCGGATTCCGGCAGCAAAATGTCGATGACTTTTATGAAATGGGAGA
GGAACTGGGCAGTGGGCAGTTTGCAATTGTGCGGAAATGCCGCGAGCGTT
CTACCGGTGTGGAGTATGCAGCCAAGTTTATTAAGAAGCGCAGATTGTCA
TCTAGTCGCCGAGGTGTGAGCCGTGAAGAAATTGAGAGGGAGGTAGACAT
ACTAAGAGAAATTCAGCACCCCAACATCATCACCTTACATGATGTGTTTG
AAAATCGCACAGATGTGGTCCTGATACTTGAGCTGGTTTCTGGAGGGGAG
CTTTTTGACTTTCTGGCACAAAAGGAAAGTTTAAGTGAGGAAGAGGCAAC
CATGTTTCTGAAGCAGATTCTTGATGGGGTTCACTATTTGCACCACAAGA
ATATTGCACATTTTGACCTCAAGCCAGAGAATATCATGCTTCTGGACCAC
AGCTCCCCCAGTCCAAGAATAAAACTTATAGATTTTGGAATTGCTCACAA
GATAGAAGCTGGGAATGAATTCAAAAATATCTTTGGAACTCCAGAATTTG
TAGCTCCTGAAATCGTAAACTATGAGCCATTAGGACTGGAAGCTGATATG
TGGAGCATTGGAGTCATCACATACATTCTCCTCAGTGGTGCATCACCATT
TCTTGGAGACACCAAGCAGGAAACTCTGACCAACATTTCAGGTGTGAATT
ATGATTTTGATGAGGAATATTTCAGCAACACTAGTGAACTGGCTAAAGAT
TTTATTCGTCGGCTATTGGTCAAAGATCCAAAAAAAAGAATGACCATTGA
TCAGAGTTTAGAGCATTCGTGGATTAAGGCTATCAAGCGTCGAAATGTCC
GACATGAGGACAGTGGAAAACGTCCTGAACGTCGTCGCCTGAAGACTACT
CGTTTGAAGGAATACACAATAAAATCACATTCTAGCATGCCCCCAAATAA
CACCTATGTGAATTTTGAAAGATTTAGCCATGTGTTGGAGGAGCTAGGAG
CAGCAGAAGATGCACTTCGTGAGCTTCAAAAGAGTCAGGCCACCTCACGC
GAGGACCTTTGCGCCCTGCGCTCTATCTATGAGGAAAAGGAAAGTTGGTA
CCATGAAGAACAGGAAGGAGTTGGGCGCCAACTAGTAGGGCTCCGGCAAG
AACTGCAGCGCAGAGAGGGTCAGCGTCGGCAGGTACAGGAGGATTTAAGG
GGAGCTATGCTTAGTGCCAATGCTCTGAAGAGAAGGTTTGGTAAACTGGA
AAATAGATATGAGGCCCTGCAGGTGCAGTTCACTGCAGAGATGAGCTTTG
TGGAAGATTTATTAAAAACTGTGGAGAGAGGTGGAGATGGATTAAGAGGG
GTTTGCTGA
>dapk3_Zebrafish gi|125806427:60-1421 PREDICTED: Danio rerio hypothetical LOC562194 (LOC562194), mRNA, 1362 bases, 9D7F7795 checksum.
ATGGCTGGCTTCAGGCAGGAGGATGTGGAGATCTACTATGACATGGGAGA
GGAGTTAGGAAGTGGCCAGTTTGCCATTGTGCGTAAATGCAAAGAAAAGA
GCAGCGGAACTGAATATGCTGCCAAGTTTATCAAGAAGAGGCGTCTGTCC
TCCAGCAGACGAGGCGTGAGCCGGGAGGAGATTGAGCGCGAGGTGAACAT
CCTTCGGGAGATTCAGCACAGCAACATCATCACACTCCATGACATCTTTG
AGAACAAGACCGATGTGATCCTGATCCTGGAGCTGGTGTCTGGAGGAGAG
CTCTTTGACTTTTTGGCTGAGAAGGAGTCACTTACTGAGGAGGAGGCCAC

GCAGTTCCTCAAGCAAATTCTGGACGGAGTTCACTACCTCCACTCTAAAC
GCATCGCTCATTTTGACTTGAAGCCTGAAAACATAATGCTGTTGGATAAA
AATGTGCCGAACCCCAGAATCAAGTTGATCGATTTTGGAATTGCACATCA
GATTAAGGATGGAAATGAATTTAAAAACATCTTTGGAACTCCTGAGTTTG
TTGCACCGGAAATTGTAAATTATGAACCCCTTGGGCTGGAGGCAGATATG
TGGAGTATTGGAGTTATCACTTATATACTCTTGAGTGGGGCTTCTCCATT
TCTTGGTGAGACCAAACAAGAAACACTTACCAATATTTCAGCAGTCAACT
ATGACTTTGACGAAGAGTACTTCAGCAACACAAGTGAACTCGCGAAGGAC
TTCATTCGGCGACTGCTCGTCAAGGATCCCAAAAAGAGAATGACAATCGA
GGACAGTCTCCAGCACTCGTGGATCAAGGTCATTAAGAGACGAAATGTTC
GACCCGAAGAGAGCGAGCGCAAGCCTGAACGCAGGCGTCTGAAGACCACC
CGTCTCAAAGAGTACACCATCAAATCCCACTCCAGCATGCCTCCCAACAA
CACCTACATCAACTTCGAGCGATTTTCCCAGGTCATGGAGGAGATCGCAG
CGGCCGAAGAAGGTCTGCGAGAGCTGGAGAAAAACCAACGATCGTGTGGC
GAGGATGTGACCGCTCTGCTCTCAATATATGAGGAGAAGGAAAGCTGGTA
CAAAGAGGAGAACGAAAGCATCGCCGCAGACCTCAGTCAGATCAAACAGG
AGCTGCAGAAAGCACAAGCCCTGCGCAGGCAGAGCCAGGAGGAGGCACGC
GCAGCCATGCTGTCAGCTAATGCCCTCAAACGCAAATTCGGACGACTGGA
GAACCGATACGAAGTTCTGGCTGAGCAGATGGACTCCGAGGTGCGCTGGG
TGGAGGAGCTGGTGCGCACCATTTCAGCTGAAAACCACACTCGCAGCACC
AGCATGGCTTGA
>dapk3_Fathead minnow
NCBI_assembled_ESTs((72422020+73556779+73634852+73512480+73721434+72761900+73439047+73721433):(122-1483)) Pimephales promelas (Fathead minnow; fish) Death-associated protein kinase 3 (DAP kinase 3; DAP-like kinase; Dlk; ZIP-kinase), 1362 bases, F4768BD7 checksum.
ATGGCTGGCTTCAGACAGGAGGATGTGGAGCTGTGCTATGAAATGGGAGA
GGAGTTAGGAAGTGGACAGTTTGCCATTGTACGTAAATGTAAAGAAAAGA
GCTCCGGAACAGAATATGCCGCCAAATTTATCAAGAAGAGACGGCTCTCA
TCCAGTCGACGGGGCGTGAGCCGAGAGGAGATCGAACGAGAGGTGAACAT
CCTCCGAGAGATTCAGCACAGCAACATTATTACCCTTCATGACATCTTTG
AGAACAAGACTGACGTGATCCTGATCCTGGAGCTGGTGTCTGGAGGGGAG
CTGTTTGACTTTCTGGCTGAGAAGGAGTCACTGACTGAGGAGGAGGCTAC
GCAGTTCCTCAAGCAGATCCTGGATGGAGTCTTCTACCTACACTCTAAAC
GCATAGCACACTTTGACTTGAAGCCTGAAAACATAATGCTGCTGGATAAA
AATGTGCCGAACCCCAGAATCAAATTGATAGATTTTGGAATTGCACATCA
GATTAAGGATGGAAACGAATTTAAAAACATCTTCGGAACTCCTGAGTTTG
TTGCACCAGAAATTGTGAATTATGAACCCCTTGGCCTGGAGGCAGACATG
TGGAGCATTGGAGTTATCACATATATACTCTTGAGTGGAGCTTCTCCGTT
TCTTGGCGAGACCAAACAAGAAACACTTACCAACATTTCAGCTGTCAACT
ATGACTTTGATGAAGAGTACTTCAGCAACACAAGTGAGCTTGCCAAGGAC
TTCATTCGCCGACTGCTGGTCAAGGATCCTAAGAAGCGAATGACAATCGA
GGACAGTCTCCAGCACCCCTGGATCAAGGTGATTAAGAGGCGCAACGTTA
GACCCGAAGAGAGCGAGCGCAAGCCGGAGCGCAGGCGTCTGAAGACCACC
CGTCTGAAGGAGTACACTATCAAATCCCACTCCAGCATGCCTCCCAAAAA
CACCTACATCAATTTCGAGCGCTTCTCTCAAGTCATGGAGGAGATCGCCG
CGGCTGAGGATGGCCTACGAGAGCTGGAGAAGAACCAGCGCTCCTGCAGG
GAGGATGTGGCCGCACTGCTATCCATCTTTGAGGAGAAGGAAAGCTGGTA

CAAGGAGGAGAACAAAAGCATCGCTGCCGATCTCAATCAGCTCAAACAGG
AGCTGCAGAAAGAACAAGCCCTGCGCAGACAGAGCCAGGAGGAGACCCGA
GCCACCATGCTGTCGGCCAATGCCCTCAAACGCAAGTTTGGTCGGTTGGA
GAACCGCTATGAAGTGCTAGCTGAGCAGATGGTCTCTGAGGTGCGCTGGG
TGGACGAGCTGGTGCGCTCAATTTCAGCTGAAAATGACACGCACAGCAAC
AGCATGGCTTAA
>dapk3_Pufferfish (47223005:(1491884-1491945+1492415-1492775+1492882-1493011+1493118-1493166+1493263-1493289+1493376-1493528+1493611-1493656+1493847-1494383)) Tetraodon nigroviridis (freshwater pufferfish) Death-associated protein kinase 3 (DAP kinase 3; DAP-like kinase; Dlk; ZIP-kinase); chromosome 15 SCAF14981, 1365 bases, 352ADE68 checksum.
ATGGCTGGCTTCAGGCAAGAGGATGTTGAGCTGTATTATGAGATGGGAGA
GGAGTTGGGCAGTGGACAGTTTGCGATTGTCCGCAAGTGCAAGGAGAAGA
GCACGGGCGTGGAATACGCCGCCAAGTTCATCAAGAAGCGGCGGCTCTCG
TCCAGCCGGCGGGGGGTGAGCCGCGAGGAGATCGAGCGCGAGGTGAACAT
CCTGAGGGAGATCCAGCACAGCAACATCATCACCCTGCACGACATCTTCG
AAAACAAGACCGACGTGATCCTGATCCTGGAACTGGTGTCCGGAGGGGAG
CTGTTTGACTTCCTGGCCGAGAAGGAGTCGCTGACGGAGGAGGAGGCCAC
GCAGTTCCTGAAGCAGATTCTGGATGGCGTCCACTACCTGCACTCCAAAC
GCATCGCGCACTTTGACCTCAAGCCCGAGAACATCATGCTGCTGGATAAA
AACGTCCCCAACCCCAGGATCAAACTGATCGATTTTGGAATCGCTCATCA
GATTAAAGCGGGAAACGAGTTTAAAAACATTTTCGGAACGCCGGAGTTTG
TCGCTCCTGAAATAGTCAACTACGAGCCGCTCGGCCTGGAGGCTGACATG
TGGAGCATTGGAGTAATTACATACATTCTGCTGAGCGGCGCGTCGCCGTT
TCTCGGCGAAACCAAGCAGGAGACCCTCACAAACATCTCAGCCGTCAACT
ACGACTTTGATGAGGAATACTTCAGCAACACCAGCGAACTGGCCAAGGAT
TTCATCCGCCGCCTGCTGGTCAAGGACCCCAAGAAGAGAATGACCATCGA
TGTCAGCTTGGAGCACCCCTGGATCAAGGTGATCAAGAGGCGAAACGTCC
GCCCAGAGGAGAGGGACCACAAGACGGAGCGCCGCCGCCTGAAGACCACT
CGTCTGAAGGAGTACACCATCAAGTCCCACTCCAGCATGCCGCCCAACAA
CACTTACATCAACTTTGAGCGCTTCTCGCAAGTGCTCGAGGAGATCGCAG
CGGCGGAGGAAGGCCTGCGGGACCTGGAGCGCAACCAGCAGTCGTGTCGG
GAAGACGTGGCAGCGCTGCTGTCCATCTACGAGGAGAAGGAGGGCTGGTA
CAAGGAGGAGAACCAGAGCATCTCCAGCGACCTGAGCCACATCCGCCGCG
AGCTGCAGCACACTCAAGCCCAGCGCAAGAAGAGCCAGGAGGAGGCGCGC
TTCACCGCGCAGGCCGCCAACGTCCTGAAGCGCAAGTTCGGGCGCTTGGA
GAACCGGTACGAGGTGCTGGCCGAGCAGGTGGCGTCGGAGGTGCGCTGGG
TGGAGGAGCTGGTCAAGTCCATGTCCATAGAGAGGGGCGGCCTCAGCCCC
AGCAGCATGCCCTGA
>dapk3_Fugu (22419072:(16803-16864+17636-17996+18117-18246+18398-18446+18542-18568+18662-18814+18894-18939+19084-19620)) Fugu rubripes (Japanese pufferfish) Death-associated protein kinase 3 (DAP kinase 3; DAP-like kinase; Dlk; ZIP-kinase), 1365 bases, 4A45E0D9 checksum.
ATGGCTGGCTTCAGGCAAGAAGATGTTGAGTTGTATTATGAAATGGGAGA
GGAGCTGGGCAGTGGGCAGTTTGCAATTGTGCGTAAGTGTAAGGAGAAGA
GCACGGGCGTGGAATACGCAGCCAAGTTCATCAAAAAGCGGCGGCTCTCC
TCCAGCCGGCGGGGCGTGAGTCGCGACGAGATCAAGCGCGAAGTGAACAT
CCTGAGGGAGATCCAGCACAGCAACATCATCACCTTGCACGACATCTTCG

AAAACAAGACCGACGTGATCCTGATCCTGGAGCTGGTGTCTGGAGGGGAG
CTGTTCGACTTCCTCGCCGAGAAGGAGTCGCTGACTGAGGAGGAGGCCAC
GCAGTTTCTGAAGCAGATCCTGGATGGCGTTCAGTATCTGCACTCCAAAC
ACATCGCACACTTTGACCTCAAGCCCGAGAACATCATGCTGCTGGATAAG
AACGTCCCTAACCCCAGGATCAAGCTGATTGATTTCGGCATTGCTCATCA
GATTAAAGCAGGAAACGAATTTAAGAATATTTTCGGAACGCCGGAGTTTG
TCGCTCCTGAAATAGTCAACTATGAGCCACTTGGACTAGAAGCTGACATG
TGGAGCATTGGAGTAATTACATACATTCTGCTGAGTGGCGCTTCGCCGTT
TCTCGGCGAAACCAAGCAGGAGACTCTCACAAACATCTCGGGCGTCAACT
ATGACTTTGATGAGGAATACTTCAGCAACACCAGTGAGCTGGCAAAAGAC
TTCATACGCCGTCTGCTGGTCAAGGACCCCAAGAAGAGAATGAAAATTGA
TGATAGCTTGGAGCACCCCTGGATCAAGGTGATTAAGAGGCGAAATGTCC
GCCCAGAGGAGAGGGACCACAAGACTGAGCGCCGCCGCCTGAAGACCACT
CGTCTGAAGGAGTACACCATCAAGTCCCACTCGAGCATGCCGCCCAACAA
CACTTATATTAACTTTGAGCGCTTCTCACAAGTGCTCGAGGAGATTGCAG
CGGCGGAGGAAGGCCTAAGGGACCTGGAACGCAACCAGCAGTCGTGTCGG
GACGATGTGGCAGCACTGTTGTCCATTTACGAGGAGAAGGAGGGGTGGTA
TAAGGAGGAGAACCAGAGCATCTCCAGTGACCTGAGCTACATCCGCCAGG
AGCTGCAACGCACCCAAATCCAGCGCAAAAAGAGCATGGAAGAGGCCCGC
TTCACCACGCAGGCTGCCAATGTCTTAAAGCGCAAGTTTGGCCGCTTGGA
GAACCGATACGAGATGCTGGCGGAGCAGGTGGCATCGGACGTCCGCTGGG
TGGAGGAGTTGGTCAAGTCCATCTCCTCAGAGAAAGCTGGCCTCGGCTCC
AGCACCATGCCCTGA
>dapk3_Medaka fish (16991872:(1422-1483+1984-2344+2568-2697+2770-2818+2904-2930+3014-3166+3355-3400+3554-4090)) Oryzias latipes (Japanese medaka fish) Death-associated protein kinase 3 (DAP kinase 3; DAP-like kinase; Dlk; ZIP-kinase), 1365 bases, 82D76130 checksum.
ATGGCTGGCTTCAGGCAGGAGGACGTGGAGCTGCATTATGAGATGGGAGA
GGAGCTGGGCAGCGGCCAGTTCGCCATCGTCCGTAAGTGCAGGGAGAAGA
GCACGGGCGGCGAGTACGCGGCCAAGTTCATCAAGAAGCGGCGTCTGTCC
TCCAGCCGGCGGGGGGTGAGCCGCGAGGAGATCGAGCGCGAGGTCAACAT
CCTGCGGGAGATCCAGCACAGCAACATCATCACCCTGCACGACATCTTCG
AGAACAAGACCGACGTGATCCTGATCCTGGAGCTGGTGTCCGGAGGAGAG
CTCTTCGACTTCCTCGCAGAGAAGGAGTCTTTGACCGAGGAGGAGGCCAC
GCAGTTCCTGAAGCAGATCCTGGACGGCGTCCAGTACCTGCACTCCAAAC
GCATCGCTCACTTCGACCTCAAGCCGGAGAACATCATGCTGCTGGACAAG
AACGTTCCCAACCCCAGGATCAAGCTGATCGACTTTGGGATCGCTCACCA
GATTAAAGCAGGAAACGAGTTCAAGAACATCTTCGGGACTCCAGAGTTTG
TGGCTCCTGAAATCGTCAACTACGAGCCGCTCGGCCTGGAGGCGGACATG
TGGAGCATCGGCGTGATCACGTACATCCTGTTGAGCGGAGCGTCTCCGTT
TCTGGGCGAGACCAAGCAGGAGACTTTGACCAACATCTCTGCCGTCAACT
ACGACTTTGACGAGGAGTATTTCAGCAACACCAGCGAGCTCGCCAAGGAC
TTCATTCGCCGCCTCCTGGTCAAGGATCCAAAGAAGAGGATGACGATTGA
CGACAGTCTAGAGCATCCCTGGATTAAGGTCATTAAGAGACGAAACGTCC
GGCCAGAGGAGGGAGACCAGAAGACCAAGCGCCGGCGCCTGAAGACCACC
CGTCTGAAGGAGTACACCATCAAGTCCCACTCCAGCATGCCCCCCAACAA
CACCTACGTCAACTTCGAGCGCTTCTCCCAGGTGCTGGAGGAGATCGCGG
CTGCAGAGGAGGGCCTGAAGGAGCTGGAGCACAACCAGCGCTCCTGCCAG

GAGGACGTGGCGGCGCTGCTGTCCATCTACGAGGAGAAGGAGGGCTGGTA
CAAGGAGGAGAACCAGAGCATCTCCTTAGACCTGAGCCACATCCGCCAAG
ACCTGCAGCGCACCCAGGCCCAGCGGAAGAAGTGCCAGGAGGAGACGCGG
CTCACCACGCAGGCCGCCAGCATCCTGAAGCGCAGGTTCGGCCGCCTGGA
GAACCGGTACGAGGTCCTGGCGGAGCAGGTGGCCTCAGAGGTGCGCTGGG
TGGAGGAGCTGGTGAAGTCCTTTTCTGAGGAGAAGGCCGCCCTGAGCCCA
GTCAGCTTGGCCTAG
>dapk3_Stickleback ((AANH01005391.1:370001-380000):(3354-3415+4304-4664+5059-5188+5374-5422+5515-5541+5661-5813+5916-5961+6385-6915)) Gasterosteus aculeatus (three spined stickleback fish) Death-associated protein kinase 3 (DAP kinase 3; DAP-like kinase; Dlk; ZIP-kinase), 1359 bases, 24AD1166 checksum.
ATGGATGGCTTCAGACAAGAGGATGTTGAGTTGTTTTATGAAATGGAAGA
GGAGTTGGGCAGTGGACAATTTGCCATCGTCCGCAAGTGTAAAGAAAAGA
GCACGAGTATCGAGTACGCAGCAAAGTTCATCAAAAAGCGGCGACTGTCA
TCCAGCCGGCGGGGGGTGAGCCGCGAGGAGATCGAGCGCGAAGTCAACAT
CCTGCGGGAGATCCAACACAGCAACATCATCACCCTGCACGACATCTTTG
AGAACAAGGCCGACGTGATCCTGATCCTGGAGCTGGTGTCCGGAGGAGAA
CTGTTTGACTTCCTGGCTGAGAAGGAATCTCTGACGGAGGAGGAGGCCAC
CCAGTTCCTCAAGCAGATCCTGGATGGCGTCCAGTACCTCCACTCCAAAC
GCATCGCTCACTTTGACCTCAAGCCTGAGAACATCATGCTGCTGGACAAG
AACGTCCCGAACCCCAGGATCAAGCTCATCGATTTTGGAATCGCGCACCA
GATAAAAGCGGGAAACGAGTTCAAGAACATATTCGGAACGCCAGAGTTTG
TCGCTCCAGAAATAGTCAACTACGAGCCGCTCGGACTGGAGGCGGACATG
TGGAGCATCGGAGTCATCACGTACATCCTACTGAGCGGCGCTTCGCCGTT
CCTGGGCGAGACTAAGCAGGAGACGCTGACCAACATCTCGGCCGTCAACT
ACGACTTCGATGAAGAGTATTTCAGTAACACCAGTGAGCTGGCAAAGGAC
TTCATACGCCGTCTGCTGGTCAAGGATCCGAAGAAGAGAATGACAATTGA
TGACAGTCTTGAGCACCCTTGGATCAAGGTTATTAAGAGGCGAAATGTCC
GCCAGGAGGAGAGAGACCACAAGACCGAGCGACGGCGCCTGAAAACCACT
CGTCTGAAGGAGTACACCATCAAGTCCCACTCCAGCATGCCCCCCAACAA
CACCTACGTCAACTTTGAGCGCTTCTCCCAGGTCCTGGAGGAGATCGCCG
CGGCAGAGGAGGGCCTGAGGGACCTGGAGCGCAACCAGCGCTCGTGCCGA
GAGGACGTGGCGGCGCTGCTGTCCATATACGAGGAGAAGGAGGGTTGGTA
CAAGGAGGAGAACCAGAGCATCTCCAGCGACCTGAGTCACATCCGCCAAG
AGCTGCAGCGCACTCAAGCCCAGCGCAAGAAGAGCCAGGAGGAGGCCCGG
CTCACCATGCAGGCCGCCAGCATCCTCAAGCGCAAGTACGGCCGCCTGGA
GAACCGCTACGAGGTCCTGGCCGAGCAAGTGGCCTCCGAAGTCCGCTGGG
TGGAGGAGCTGGTCAAGTCCATGTCGGCGGAGAGGAGCGGCCTCGGCAGC
ATGGCCTGA
>dapk3_ Astatotilapia 46489038:1-550 Astatotilapia burtoni (Cichlid fish) cDNA clone hh_Ab_Brain2000_000004668 5', mRNA Death-associated protein kinase 3 (DAP kinase 3; DAP-like kinase; Dlk; ZIP-kinase); partial EST missing N-part, 550 bases, 3BC9646F checksum.
GCCAGGAGGACAGAGATCACAAGACTGAGCGCCGGCGCCTGAAGACTACT
CGTCTCAAGGAGTATACCATCAAGTCCCACTCCAGCATGCCTCCGAACAA
CACCTACGCCAACTTTGAGCGCTTCTCCCAGGTGCTCGAGGAGATTGCGG
CGGCTGAGGAAGGCCTGAAGGAGTTGGAGCGCAACCAGCGCTCTTGCCGC
GAGGACGTGGCAGCGCTGCTATCGATATATGAGGAGAAGGAGGGGTGGTA

Fig. S3

CAAGGAAGAGAACCAGAGCATCTCTGGTGACCTGAACCACATCCGCCAAG
AGCTGCAGCGCACGCAGACGCAGCGCAAGAAGTGCCAGGAGGACGCGCGG
GTCACTATGCAGTCGGCCAACATCCTCAAGCGCAAGTTTGGGCGCCTGGA
GAACCGCTACGAGGTCCTGGCCGAGCAGGTGGCCTCCGAGGTCCGCTGGG
TGGAGGAGCTGGTGAAGTCAATATCGGCAGAGAAGGACGGCCTCAGCTCT
GGCAGCACGCCGTGAGCGGCGCTGACATCCAGCAGTGAGGGTTTCATCAT
