# Supplementary material for: ZIPK: A Unique Case of Murine-Specific Divergence of a Conserved Vertebrate Gene
Source: PLoS Genet. 2007 Oct 19;3(10):e180. doi: 10.1371/journal.pgen.0030180 (PMC2041995; doi:10.1371/journal.pgen.0030180)
Supplement: Supplementary file 3 [file pgen.0030180.sd001.doc]

Accession numbers:

NCBI GI numbers of the ZIPk sequences used:

*Homo sapiens* 4557511

*Pan troglodytes* 114674687

*Macaca mulatta* 109122939

*Canis familiaris* 73987437

*Bos taurus* 76622257

*Mus musculus* 6681133

*Rattus norvegicus* 11968142

*Xenopus laevis* 66911521

*Xenopus tropicalis* 62860094

*Tetraodon nigroviridis* 47223108

*Danio rerio* 68356496

Sequences of the following ZIPk were extracted by us from genomic contigs, ESTs, or assembled raw sequencing traces (the latter indicated by TI, and available at the NCBI trace archive database at <http://www.ncbi.nlm.nih.gov/Traces>). Assembly was done with the CAP3 program [21]. When needed sequences from different sources for the same organism were combined. The coding regions on the genomic contigs are detailed in the supplementary material.

Tupaia belangeri 108226212+107812812;

Cavia porcellus: TI|(812701167+816150362) + TI|(1589066801+816143923+1588856408+812549142+1586592910+760990457+1593835765+1587132918) + TI|1583699030 + 91731898;

Dipodomys ordii: TI|(1542302641+1541211393+1578971935+1562912636+1552706462) + TI|(1569549916+1566271599+1585076393+1585061768+1542035913+1556201828+1541200922+1586154871+1585648556+1535177412) + TI|1587085697 + TI|1569544450

Myotis lucifugus: TI|(976759098+964301557+981846460) + 105860175;

Sorex araneus: 80402909 + 80402905 + TI|(838222606+892417228) + TI|(873967520+875830371+894062723+845045749+848969792);

Monodelphis domestica: 84819837;

Ornithorhynchus anatinus: 91473547;

Gallus gallus: 51039807 + 25936497 + 25365707 +TI|227294367;

Taeniopygia guttata: TI|(1401310278+1277343563+1253220430+1397767862 +1249786453+1290200497+1398826774+1397636686+1423694077) + TI|(1242613131+1422732028+1404775456+127731875 1) + TI|1241607171;

Pimephales promelas: 72422020 + 73556779 + 73634852 + 73512480 + 73721434 + 72761900 + 73439047 + 73721433;

Fugu rubripes: 22419072;

Oryzias latipes: 16991872;

Gasterosteus aculeatus: 86297297;

Astatotilapia burtoni: 46489038.
