# Supplementary material for: ZIPK: A Unique Case of Murine-Specific Divergence of a Conserved Vertebrate Gene
Source: PLoS Genet. 2007 Oct 19;3(10):e180. doi: 10.1371/journal.pgen.0030180 (PMC2041995; doi:10.1371/journal.pgen.0030180)
Supplement: Supplementary file 4 [file pgen.0030180.sg002.doc]

> Homo sapiens (Human)

Fig. S2

MSTFRQEDVEDHYEMGEELGSGQFAIVRKCRQKGTGKEYAAKFIKKRRLS

SSRRGVSREEIEREVNILREIRHPNIITLHDIFENKTDVVLILELVSGGE

LFDFLAEKESLTEDEATQFLKQILDGVHYLHSKRIAHFDLKPENIMLLDK

NVPNPRIKLIDFGIAHKIEAGNEFKNIFGTPEFVAPEIVNYEPLGLEADM

WSIGVITYILLSGASPFLGETKQETLTNISAVNYDFDEEYFSNTSELAKD

FIRRLLVKDPKRRMTIAQSLEHSWIKAIRRRNVRGEDSGRKPE-RRRLKT

TRLKEYTIKSHSSLPPNNSYADFERFSKVLEEAAAAEEGLRELQRSRRLC

HEDVEALAAIYEEKEAWYREESDSLGQDLRRLRQELLKTEALKRQAQEEA

KGALLGTSGLKRRFSRLENRYEALAKQVASEMRFVQDLVRALEQEKLQGV

ECGLR--

> Pan troglodytes (Chimpanzee)

MSTFRQEDVEDHYEMGEELGSGQFAIVRKCRQKGTGKEYAAKFIKKRRLS

SSRRGVSREEIEREVNILREIRHPNIITLHDIFENKTDVVLILELVSGGE

LFDFLAEKESLTEDEATQFLKQILDGVHYLHSKRIAHFDLKPENIMLLDK

NVPNPRIKLIDFGIAHKIEAGNEFKNIFGTPEFVAPEIVNYEPLGLEADM

WSIGVITYILLSGASPFLGETKQETLTNISAVNYDFDEEYFSNTSELAKD

FIRRLLVKDPKRRMTIAQSLEHSWIKAIRRRNVRGEDSGRKPE-RRRLKT

TRLKEYTIKSHSSLPPNNSYADFERFSKVLEEAAAAEEGLRELQRSRRLC

HEDVEALAAIYEEKEAWYREESDSLGQDLRRLRQELLKTEALKRQAQEEA

KGALLGTSGLKRRFSRLENRYEALAKQVASEMRFVQDLVRALEQEKLQGV

ECGLR--

> Macaca mulatta (Macaque)

MSTFRQEDVEDHYEMGEELGSGQFAIVRKCRQKGTGKEYAAKFIKKRRLS

SSRRGVSREEIEREVNILREIRHPNIITLHDIFENKTDVVLILELVSGGE

LFDFLAEKESLTEDEATQFLKQILDGVHYLHSKRIAHFDLKPENIMLLDK

NVPNPRIKLIDFGIAHKIEAGNEFKNIFGTPEFVAPEIVNYEPLGLEADM

WSIGVITYILLSGASPFLGETKQETLTNISAVNYDFDEEYFSNTSELAKD

FIRRLLVKDPKQRMTIAQSLEHSWIKAIRRRNVRGEDSGRKPE-RRRLKT

TRLKEYTIKSHSSMPPNNSYANFERFSKVLEEVAAAEEGLRELERSRRLC

HEDVEALAAIYEEKEAWYREESDSLGQDLRRLRQELLKTEALKRQAQEEA

KGALLGTSGLKRRFSRLENRYEALAKQVASEMRFVQDLVRALEQEKLQGV

ECGLR--

> Rattus norvegicus (Rat)

MSTFRQEDVEDHYEMGEELGSGQFAIVRKCQQKGTGMEYAAKFIKKRRLP

SSRRGVSREEIEREVSILREIRHPNIITLHDVFENKTDVVLILELVSGGE

LFDFLAEKESLTEDEATQFLKQILDGVHYLHSKRIAHFDLKPENIMLLDK

HAASPRIKLIDFGIAHRIEAGSEFKNIFGTPEFVAPEIVNYEPLGLEADM

WSIGVITYILLSGASPFLGETKQETLTNISAVNYDFDEEYFSSTSELAKD

FIRRLLVKDPKRRMTIAQSLEHSWIKV-RRR----EDGARKPE-RRRLRA

ARLREYSLKSHSSMPRNTSYASFERFSRVLEDVAAAEQGLRELQRGRRQC

RERVCALRVAAEQREARCRDGSAGLGRDLRRLRTELGRTEALRTRAQEEA

RAALLGAGGLKRRLCRLENRYDALAAQVAAEVQFVRDLVRALEQERLQAE

CGVR---

Fig. S2

> Mus musculus (Mouse)

MSTFRQEDVEDHYEMGEELGSGQFAIVRKCQQKGTGMEYAAKFIKKRRLP

SSRRGVSREEIEREVSILREIRHPNIITLHDVFENKTDVVLILELVSGGE

LFDFLAEKESLTEDEATQFLKQILDGVHYLHSKRIAHFDLKPENIMLLDK

HAASPRIKLIDFGIAHRIEAGSEFKNIFGTPEFVAPEIVNYEPLGLEADM

WSIGVITYILLSGASPFLGETKQETLTNISAVNYDFDEEYFSSTSELAKD

FIRRLLVKDPKRRMTIAQSLEHSWIKV-RRR----EDGARKPE-RRRLRA

ARLREYSLKSHSSMPRNTSYASFERFSRVLEDVAAAEQGLRELQRGRRQC

RERVCALRAAAEQREARCRDGSAGLGRDLRRLRTELGRTEALRTRAQEEA

RAALLGAGGLKRRLCRLENRYDALAAQVAAEVQFVRDLVRALEQERLQAE

CGVR---

> Dipodomys ordii (Kangaroo rat)

xxxxxxxxxxxxxxxxxxxxxGQFAIVRKCRQKGTGKEYAAKFIKKRRLS

SSRRGVSREEIEREVSILREIRHPNIITLHDVFENKTDVVLILELVSGGE

LFDFLAEKESLTEDEATQFLKQILDGVHYLHSKRIAHFDLKxxxxxxxxx

xxxxxxxxxIDFGIAHKIEAGNEFKNIFGTPEFVAPEIVNYEPLGLEADM

WSIGVITYILLSGASPFLGETKQETLTNISAVNYDFDEEYFSSTSELAKD

FIRRLLVKDPKRRMTIAQSLEHSWIKAIRRRAVRSEDGGRRAE-RRRLRT

TRLKEYTXXXHSSMPPNNSYANFERFSKVLEEVAAAEEGLRELERGRRLC

REDVEALAAIYEEKEAWYREESAGLGQDVRRLRQELHRADALRRQAHEEV

RAALLGSGxxxxxxxxxxxxxxxxxxxxxxxxxxxxxxxxxxxxxxxxxx

xxxxx--

> Cavia porcellus (Cavia)

xxxxxxEDVEDHYEMGEELGSGQFAIVRKCRQKGTGREYAAKFIKKRRLS

SSRRGVSREEIEREVSILREIRHPNIITLHDVFENKTDVVLILELVSGGE

LFDFLAEKESLTEDEATQFLKQILDGVHYLHSKRIAHFDLKPENIMLLDK

SVPSPHIKLIDFGIAHKIEAGNEFKNIFGTPEFVAPEIVNYEPLGLEADM

WSIGVITYILLSGASPFLGETKQETLTNISAVNYDFDEEYFSNTSELAKD

FIRRLLVKDPKRRMTIAQSLEHSWIKAIRRRNVRGEDGGRRPE-RRRLRA

ARLKEYTIKSHSSMPPNNSYASFERFSRVLEEVAAAEEGLRELQRGRRLC

REDVEALAAIYEEKEAWYTEERDSIGRDLRRLRQELHKTEALQRQAQEEA

KGALLGASGLKRRFSRLENRYEALAKQVASEMRFVQELVRALEQERLQGG

ECGLR--

> Tupaia belangeri (Tupaia) xxxxxxxxxxxxxxxxxxxxxGQFAIVRKCRQKGTGKEFAAKFIKKRRLS

SSRRGVSREEIEREVDILREIRHPNIIALHDIFENRTDVVLILELVSGGE

LFDFLAEKESLTEDEATQFLKQILDGVHYLHSKRIAHFDLKPENIMLLDK

DVPNPRIKLIDFGIAHKIEAGNEFKNIFGTPEFVAPEIVNYEPLGLEADM

WSIGVITYILLSGASPFLGETKQETLTNISAVNYDFDEEYFGNTSELAKD

FIRRLLVKDPKRRMTIAQSLEHSWIKAIRRRNVRSEDSGRKPE-RRRLKT

Fig. S2

ARLKEYTIKSHSSMPPNNTYVNFERFSKVLEEVAAAEEGLRELERSRRLC

HEDVAALTAIYEEKEAWYREEHESIGQDLRRLRQELHKTEALKRQAQEEA

KGALLGAGGLRRRLSRLENRYEALAKQVASEMRFVQDLVRAMEQEKLQAV

GCGLR--

> Canis familiaris (Dog)

MSTFRQEDVEDYYEMGEELGSGQFAIVRKCRQKGTGKEYAAKFIKKRRLS

SSRRGVSREEIEREVNILREIRHPNIITLHDIFENKTDVVLILELVSGGE

LFDFLAEKESLTEDEATQFLKQILDGVHYLHSKRIAHFDLKPENIMLLDK

NVPNPRIKLIDFGIAHKIEAGNEFKNIFGTPEFVAPEIVNYEPLGLEADM

WSIGVITYILLSGASPFLGETKQETLTNISAVNYDFDEEYFSNTSELAKD

FIRRLLVKDPKRRMTIAQSLEHSWIKAIRRRNVRREDSGRKPE-RRRLKT

ARLKEYTIKSHSSMPPNNTYVNFERFSKVLEEVAAAEEGLRGLEHSRRLF

HEDIEALTAIYEEKEAWYREENESIGQDLRRLRQELHKTEALQRQAQEEA

KGALLGASGLKRRFSRLENRYEALAKQVASEMRFVQDLVRAMEQEKLQGV

ECGLR--

> Bos Taurus (Cow)

MSTFRQEDVEDHYEMGEELGSGQFAIVRKCRQKGTGKEYAAKFIKKRRLS

SSRRGVSREEIEREVNILREIRHPNIITLHDIFENRTDVVLILELVSGGE

LFDFLAEKESLTEDEATQFLKQILDGVHYLHSKRIAHFDLKPENIMLLDK

NVPNPRIKLIDFGIAHKIEAGNEFKNIFGTPEFVAPEIVNYEPLGLEADM

WSIGVITYILLSGASPFLGETKQETLTNISAVNYDFDEEYFSNTSELAKD

FIRRLLVKDPKRRMTIAQSLEHSWIKAIRRRNVRREDSGRKPE-RRRLKT

ARLKEYTIKSHSSMPPNNTYINFERFSKVLEEVAAAEEGLRGLEHSRRLF

HEDLEALTAIYEEKEAWYREENESLGQDLRRLRQELHKTEALQRQAQEEA

KGALLGASGLKRRFSRLENRYEALAKQVASEMRFVQDLVRAMEQEKLQEG

ECSLR--

> Myotis lucifugus (Bat)

xxxxxxxxxxxxxxxxxxxxxGQFAIVRKCRQKGTGKEYAAKFIKKRRLS

SSRRGVSREEIEREVNILREIRHPNIITLHDIFENKTDVVLILELVSGGE

LFDFLAEKESLTEDEATQFLKQILDGVHYLHSKRIAHFDLKPENIMLLDX

NVPNPRIKLIDFGIAHKIEAGNEFKNIFGTPEFVAPEIVNYEPLGLEADM

WSIGVITYILLSGASPFLGETKQETLTNISAVNYDFDEEYFSNTSELAKD

FIRRLLVKDPKRRMTISQSLEHSWIKAIRRRNVRREDSGRKPE-RRRLKT

ARLKEYTIKSHSSMPPNNTYINFERFSKVLEEVAAAEEGLRGLEHSRRLF

HEDIEALTAIYEEKEAWYREENESIGQDLRRLRQELHKTEALQRQAQEEA

KGALLGASGLKRRFSRLENRYEALAKQVASEMRFVQDLVRAMEQEKQQEV

ECGLR--

> Sorex araneus (Shrew)

xxxxxxxxxxxxxxxxxxxxxxxxxxVRKCRXXGTGKEYAxxxxxKRRLS

SSRRGVSREEIEREVDILREIRHPNIITLHDVFENKTDVVLILELVSGGE

LFDFLAEKESLTEDEATQFLKQILDGVHYLHSKRIAHFDLKPENIMLLDK

NVASPRIKLIDFGIAHKIEAGNEFKNIFGTPEFVAPEIVNYEPLGLEADM

Fig. S2

WSIGVITYILLSGASPFLGDTKQETLTNISAVNYDFDEEYFSSTSELAKD

FIRRLLVKDPKRRMTIAESLEHSWIKAIRRRNVRREDSGKKPE-RRRLKT

ARLKEYTIKSHSSMPPNHTYINFERFSKVLEEVAAAEEGLRGLEHSRRLC

HEDVEALTAIYEEKEAWYREEQEGLGQDLRRLRQELHKTETLRRQAQEEA

KGALLGANGLKRRFSRLENRYEALAQQLASEMRFVQDLVRTMEQEKLQGG

QCGLR--

> Monodelphis domestica (Opossum)

MSTFRQEDVEDHYEMGEELGSGQFAIVRKCRQKSNGMEYAAKFIKKRRLS

SSRRGVSREEIEREVDILREIQHPNIITLHDIFENKTDVVLILELVSGGE

LFDFLAEKESLTEEEATQFLKQILDGVHYLHSKRIAHFDLKPENIMLLDK

NVPNPRIKLIDFGIAHKIEAGNEFKNIFGTPEFVAPEIVNYEPLGLEADM

WSIGVITYILLSGASPFLGETKQETLTNISAVNYDFDEEYFSNTSELAKD

FIRRLLVKDPKKRMTIDQSLEHSWIKVIKRRNVRNEDSGKKPE-RRRLKT

TRLKEYTIKSHSSMPPNNTYINFERFSKVLEEVAAAEEGLRELERSKKLF

REDIEVLTSIYEEKEAWYKEENESIGQDLRLLRQELHKTEALKRQAQEEA

KGALLGANGLKRRFSRLENRYEALVKQVASEMRFVQDLVYAMEQEKMQDG

DCSVR--

> Ornithorhynchus anatinus (Platypus) xxxxxxxxxxxxxxxxxxxxxGQFAIVRKCRQKSNGLEYAAKFIKKRRLS

SSRRGVSREEIEREVDILREIQHPNIITLHDIFENKTDVVLILELVSGGE

LFDFLAEKESLTEEEATQFLKQILDGVHYLHSKHIAHFDLKPENIMLLDK

NVPNPRIKLIDFGIAHKIEAGNEFKNIFGTPEFVAPEIVNYEPLGLEADM

WSIGVITYILLSGASPFLGETKQETLTNISAVNYDFDEEYFSNTSELAKD

FIRRLLVKDPKKRMTIAQSLEHSWIKVRRRRNVRNEDSGKKPE-RRRLKT

TRLKEYTIKSHSSMPPNNTYINFERFSKVLEEVAAAEEGLRELEHSKKLF

REDIEALTSIYEEKEAWYKEENESIGQDLRQLRQELHKTEALKRQTQEEA

KGALLAANGLKRGFGRLENRFEALAKQVASEMRFVQDLVRAMEQEKLQES

DCSIR--

> Xenopus laevis

MSGFRQQNVDDFYEMGEELGSGQFAIVRKCRERSTGVEYAAKFIKKRRLS

SSRRGVSREEIEREVDILREIQHPNIITLHDVFENRTDVVLILELVSGGE

LFDFLAQKESLSEEEATMFLKQILDGVHYLHHKNIAHFDLKPENIMLLDQ

SSPSPRIKLIDFGIAHKIEAGNEFKNIFGTPEFVAPEIVNYEPLGLEADL

WSIGVITYILLSGASPFLGDTKQETLTNISGVNYDFDEEYFSNTSELAKD

FIRRLLVKDPKKRMTIDQSLEHSWIKAIKRRNVRHEDSGKRPE-RRRLKT

TRLKEYTIKSHSSMPPNNTYVNFERFSHVLEELGAAEDALRELQKSQATS

REDLCALRSIYEEKESWYHEEQEGVGRQLVGLRQELQRRESQRRQVQEDL

RGAMLSANALKRRFGKLENRYEALQAQFTAEMSFVEDLLKTVERGEDGLR

GVC---

> Xenopus tropicalis

MSGFRQQNVDDFYEMGEELGSGQFAIVRKCRERSTGVEYAAKFIKKRRLS

Fig. S2

SSRRGVSREEIEREVDILREIQHPNIITLHDVFENRTDVVLILELVSGGE

LFDFLAQKESLSEEEATMFLKQILDGVHYLHHKNIAHFDLKPENIMLLDH

SSPSPRIKLIDFGIAHKIEAGNEFKNIFGTPEFVAPEIVNYEPLGLEADM

WSIGVITYILLSGASPFLGDTKQETLTNISGVNYDFDEEYFSNTSELAKD

FIRRLLVKDPKKRMTIDQSLEHSWIKAIKRRNVRHEDSGKRPE-RRRLKT

TRLKEYTIKSHSSMPPNNTYVNFERFSHVLEELGAAEDALRELQKSQATS

REDLCALRSIYEEKESWYHEEQEGVGRQLVGLRQELQRREGQRRQVQEDL

RGAMLSANALKRRFGKLENRYEALQVQFTAEMSFVEDLLKTVERGGDGLR

GVC----

> Gallus gallus (Chicken)

MSTFRQESVEDFYEMGEELGHGQFAIVRKCRERKTGVEYAAKFIKKRRLS

SSRRGVSREEIEREVDILREIQHPNIITLHDIFENKTDVVLILELVSGGE

LFDFLAEKESLTEEEATQFLKQILDGVHYLHSKRIAHFDLKPENIMLLDK

NVPNPRIKLIDFGIAHKIEAGNEFKNIFGTPEFVAPEIVNYEPLGLEADM

WSIGVITYILLSGASPFLGETKQETLTNISAVNYDFDEEYFSSTSELAKD

FIRRLLVKDPKKRMTIAQSLEHPWIKVIKRRNVRNEDSGKKAEqRRRLKT

TRLKEYTIKSHSSMPPNNTYVNFERFSKVMEEVAAAEETLREIQRNKRSF

REDIEALLSIYEEKESWYKEENESIGRDLRQIRQELLRAESARQRAQEES

RNVMQAAGGLRRRYRRLEKRYGALAEQVASEVRFVQELLWSIEREKLQSG

GEDGSIR

> Taeniopygia guttata (Finch)

MSTSARRSVEDFYEMGEELGSGQFAIVRKCRERKTGLEYAAKFIKKRRLS

SSRRGVSREEIEREVDILREIQHPNIITLHDIFENKTDVVLILELVSGGE

LFDFLAEKESLTEEEATQFLKQILDGVHYLHSKRIAHFDLKPENIMLLDK

NVPNPRIKLIDFGIAHKIEAGNEFKNIFGTPEFVAPEIVNYEPLGLEADM

WSIGVITYILLSGASPFLGETKQETLTNISAVNYDFDEEYFSNTSELAKD

FIRRLLVKDPKKRMTIAQSLEHPWIKVIKRRNVRNEDSCKKPE-RRRLKT

TRLKEYTIKSHSSMPPNNTYINFERFSKVMEEVAAAEESLRELERNKKSF

QEDIEALLSIYEEKESWYKEENESISQDLRQIRQELQKTEALKKQAQEET

KSVVQAANGLKRRYRKLENHYEALAKQVASEMKFVQELVWSIEREKLQSS

EGDGSIR

> Danio rerio (Zebrafish)

MAGFRQEDVEIYYDMGEELGSGQFAIVRKCKEKSSGTEYAAKFIKKRRLS

SSRRGVSREEIEREVNILREIQHSNIITLHDIFENKTDVILILELVSGGE

LFDFLAEKESLTEEEATQFLKQILDGVHYLHSKRIAHFDLKPENIMLLDK

NVPNPRIKLIDFGIAHQIKDGNEFKNIFGTPEFVAPEIVNYEPLGLEADM

WSIGVITYILLSGASPFLGETKQETLTNISAVNYDFDEEYFSNTSELAKD

FIRRLLVKDPKKRMTIEDSLQHSWIKVIKRRNVRPEESERKPE-RRRLKT

TRLKEYTIKSHSSMPPNNTYINFERFSQVMEEIAAAEEGLRELEKNQRSC

GEDVTALLSIYEEKESWYKEENESIAADLSQIKQELQKAQALRRQSQEEA

RAAMLSANALKRKFGRLENRYEVLAEQMDSEVRWVEELVRTISAENHTRS

TSMA---

Fig. S2

> Pimephales promelas (Fathead minnow)

MAGFRQEDVELCYEMGEELGSGQFAIVRKCKEKSSGTEYAAKFIKKRRLS

SSRRGVSREEIEREVNILREIQHSNIITLHDIFENKTDVILILELVSGGE

LFDFLAEKESLTEEEATQFLKQILDGVFYLHSKRIAHFDLKPENIMLLDK

NVPNPRIKLIDFGIAHQIKDGNEFKNIFGTPEFVAPEIVNYEPLGLEADM

WSIGVITYILLSGASPFLGETKQETLTNISAVNYDFDEEYFSNTSELAKD

FIRRLLVKDPKKRMTIEDSLQHPWIKVIKRRNVRPEESERKPE-RRRLKT

TRLKEYTIKSHSSMPPKNTYINFERFSQVMEEIAAAEDGLRELEKNQRSC

REDVAALLSIFEEKESWYKEENKSIAADLNQLKQELQKEQALRRQSQEET

RATMLSANALKRKFGRLENRYEVLAEQMVSEVRWVDELVRSISAENDTHS

NSMA---

> Tetraodon nigroviridis (Pufferfish)

MAGFRQEDVELYYEMGEELGSGQFAIVRKCKEKSTGVEYAAKFIKKRRLS

SSRRGVSREEIEREVNILREIQHSNIITLHDIFENKTDVILILELVSGGE

LFDFLAEKESLTEEEATQFLKQILDGVHYLHSKRIAHFDLKPENIMLLDK

NVPNPRIKLIDFGIAHQIKAGNEFKNIFGTPEFVAPEIVNYEPLGLEADM

WSIGVITYILLSGASPFLGETKQETLTNISAVNYDFDEEYFSNTSELAKD

FIRRLLVKDPKKRMTIDVSLEHPWIKVIKRRNVRPEERDHKTE-RRRLKT

TRLKEYTIKSHSSMPPNNTYINFERFSQVLEEIAAAEEGLRDLERNQQSC

REDVAALLSIYEEKEGWYKEENQSISSDLSHIRRELQHTQAQRKKSQEEA

RFTAQAANVLKRKFGRLENRYEVLAEQVASEVRWVEELVKSMSIERGGLS

PSSMP--

> Fugu rubripes (Fugu)

MAGFRQEDVELYYEMGEELGSGQFAIVRKCKEKSTGVEYAAKFIKKRRLS

SSRRGVSRDEIKREVNILREIQHSNIITLHDIFENKTDVILILELVSGGE

LFDFLAEKESLTEEEATQFLKQILDGVQYLHSKHIAHFDLKPENIMLLDK

NVPNPRIKLIDFGIAHQIKAGNEFKNIFGTPEFVAPEIVNYEPLGLEADM

WSIGVITYILLSGASPFLGETKQETLTNISGVNYDFDEEYFSNTSELAKD

FIRRLLVKDPKKRMKIDDSLEHPWIKVIKRRNVRPEERDHKTE-RRRLKT

TRLKEYTIKSHSSMPPNNTYINFERFSQVLEEIAAAEEGLRDLERNQQSC

RDDVAALLSIYEEKEGWYKEENQSISSDLSYIRQELQRTQIQRKKSMEEA

RFTTQAANVLKRKFGRLENRYEMLAEQVASDVRWVEELVKSISSEKAGLG

SSTMP--

> Oryzias latipes (Medaka-fish)

MAGFRQEDVELHYEMGEELGSGQFAIVRKCREKSTGGEYAAKFIKKRRLS

SSRRGVSREEIEREVNILREIQHSNIITLHDIFENKTDVILILELVSGGE

LFDFLAEKESLTEEEATQFLKQILDGVQYLHSKRIAHFDLKPENIMLLDK

NVPNPRIKLIDFGIAHQIKAGNEFKNIFGTPEFVAPEIVNYEPLGLEADM

WSIGVITYILLSGASPFLGETKQETLTNISAVNYDFDEEYFSNTSELAKD

FIRRLLVKDPKKRMTIDDSLEHPWIKVIKRRNVRPEEGDQKTK-RRRLKT

TRLKEYTIKSHSSMPPNNTYVNFERFSQVLEEIAAAEEGLKELEHNQRSC

QEDVAALLSIYEEKEGWYKEENQSISLDLSHIRQDLQRTQAQRKKCQEET

RLTTQAASILKRRFGRLENRYEVLAEQVASEVRWVEELVKSFSEEKAALS

Fig. S2

PVSLa--

> Gasterosteus aculeatus (Stickleback)

MDGFRQEDVELFYEMEEELGSGQFAIVRKCKEKSTSIEYAAKFIKKRRLS

SSRRGVSREEIEREVNILREIQHSNIITLHDIFENKADVILILELVSGGE

LFDFLAEKESLTEEEATQFLKQILDGVQYLHSKRIAHFDLKPENIMLLDK

NVPNPRIKLIDFGIAHQIKAGNEFKNIFGTPEFVAPEIVNYEPLGLEADM

WSIGVITYILLSGASPFLGETKQETLTNISAVNYDFDEEYFSNTSELAKD

FIRRLLVKDPKKRMTIDDSLEHPWIKVIKRRNVRQEERDHKTE-RRRLKT

TRLKEYTIKSHSSMPPNNTYVNFERFSQVLEEIAAAEEGLRDLERNQRSC

REDVAALLSIYEEKEGWYKEENQSISSDLSHIRQELQRTQAQRKKSQEEA

RLTMQAASILKRKYGRLENRYEVLAEQVASEVRWVEELVKSMSAERSGLG

sma----

> Astatotilapia burtoni (Astatotilapia)

xxxxxxxxxxxxxxxxxxxxxxxxxxxxxxxxxxxxxxxxxxxxxxxxxx

xxxxxxxxxxxxxxxxxxxxxxxxxxxxxxxxxxxxxxxxxxxxxxxxxx

xxxxxxxxxxxxxxxxxxxxxxxxxxxxxxxxxxxxxxxxxxxxxxxxxx

xxxxxxxxxxxxxxxxxxxxxxxxxxxxxxxxxxxxxxxxxxxxxxxxxx

xxxxxxxxxxxxxxxxxxxxxxxxxxxxxxxxxxxxxxxxxxxxxxxxxx

xxxxxxxxxxxxxxxxxxxxxxxxxxxxxxxxxxQEDRDHKTE-RRRLKT

TRLKEYTIKSHSSMPPNNTYANFERFSQVLEEIAAAEEGLKELERNQRSC

REDVAALLSIYEEKEGWYKEENQSISGDLNHIRQELQRTQTQRKKCQEDA

RVTMQSANILKRKFGRLENRYEVLAEQVASEVRWVEELVKSISAEKDGLS

SGSTP--
