# Supplementary material for: ZIPK: A Unique Case of Murine-Specific Divergence of a Conserved Vertebrate Gene
Source: PLoS Genet. 2007 Oct 19;3(10):e180. doi: 10.1371/journal.pgen.0030180 (PMC2041995; doi:10.1371/journal.pgen.0030180)
Supplement: Supplementary file 5 [file pgen.0030180.sg006.doc]

PAR-4 Multiple Sequence Alignment

Fig. S6

```
Mouse     GPSARKGKGQIEKRKLREKRRSTGVVNIPAAECLDEYEDDEAGQKERKREDAITQQNTIQ   193

Rat       GPSARKGKGQIEKRKLREKRRSTGVVNIPAAECLDEYEDDEAGQKERKREDAITQQNTIQ   192

Human     GPSARKGKGQIEKRKLREKRRSTGVVNIPAAECLDEYEDDEAGQKERKREDAITQQNTIQ   200

Chimpanze GPSARKGKGQIEKRKLREKRRSTGVVNIPAAECLDEYEDDEAGQKERKREDAITQQNTMQ   200

Macaque   GPSARKGKGQIEKRKLREKRRSTGVVNIPAAECLDEYEDDEAGQKERKREDAITQQNTMQ   200

Cow       GPSARKGKGQIEKRKLREKRRSTGVVNIPAAECLDEYEDDEAGQKERKREDAITQQNTMQ   121

Horse     GPSSRKGKGQIEKRXXXXXXXXXXXXXXXXXXCLDEYEDDEAGQKERKREDAITQQNTMQ    73

Dog       --------------------------------CLDEYEDDEAGQKERKREDAITQQNTMQ    28

Shrew     DPSARKGKAQMEKSKLXXXXXSTGVVNIPAAECLDEYEDDEMGQNERKREDAITQPNTVQ    76

Opossum   GPSARKGKGQIEKRKLREKRRSTGVVNIPAAECLDEYEDDEAGQKERKREDAITQQNTMQ   201

Chicken   --------------------------------SLDEYEDDEAGQKERKKEDAITQQNTIQ    71

Xenopus_l GPSARKGKGQIEKRKLREKRRSTGVVNIPSVESPDEYEDDEAGQKARKGENVVTKQNTLQ   208

Zebrafish GPSARKGKGQIEKRKLREKRRSTGVVSIPSNESLDELDDDDGEEKERKMEENLTQHNTVQ   185

Tetradon  GPSARKGKGQIEKRKLREKRRSTGVVSIPSNESLDELDDDDPGERERREEEALVQANTYQ   204

                                          ============================
```

Fig. S6

```
Mouse     NEAATLPDPGtSYLPQDPSRTVPG-------RYKStTSAPEDEISNRYPRTDRSGFsRHN   246

Rat       NEAASLPDPGtSYLPQDPSRTVPG-------RYKStISAPEEEILNRYPRTDRSGFsRHN   245

Human     NEAVNLLDPGsSYLLQEPPRTVSG-------RYKStTSVSEEDVSSRYSRTDRSGFpRYN   253

Chimpanze NEAVNLLDPGsSYLVQEPPRTVSG-------RYKStTSVSEEDVSSRYSRTDRSGFpRYN   253

Macaque   NEAVNLLDPGsSYLLQEPPRTVSG-------RYKStTSVSEEDVSSRYSRTDRSGFpRYN   253

Cow       NEAVSLLDPGsSYLLQEPSRTVSG-------RYKStTTASEEDVSSRFPRTDRSGFnRYN   174

Horse     NEAVSLLEPGsSYLLQEPSRTVSG-------RYKStTTASEEDVSSRYPRTDRSGFsRYN   126

Dog       NEAVSLLDPGsSYLLQESSRTVSG-------RYKS-TTASEEDVSSRYPRTDRSGFsRYN    80

Shrew     NEAVSLLDPGsCYLLQEPSGTLSG-------RYKSaTTGPEGEILSRYPRTERSGFrRYN   129

Opossum   NEAATLDPGG-SYLLQEPSRTVSS-------RYKStSITPEDDVSSRYSRIDRGGF-RYN   252

Chicken   NESSSadstg-SYLIQDSSRMVSS-------RYKStGNAPGDDALNRYSLTERTTYsRYS   123

Xenopus_l NESFSldpse-SFLPQETVKTAGSn------RYKS-NNAPDDDILNHFSRNDRTAFnHLN   260

Zebrafish NEALTsehea-AHSVQDNSRSRSHksdeegsRHRH----------------------RHG   222

Tetradon  NEAMTaesn--PERLPDTPRSGSS-------RHKS-CVGAEEEVGGSRQWPQQWPQ-RHA   253

          =====      =============       ====                      ===
```

Fig. S6

```
Mouse     RDANAPASFSSSST-LEKRIEDLEKEVVRERQENLRLVRLMQDKEEMIGKLKEEIDLLNR   305

Rat       RDTSAPANFASSST-LEKRIEDLEKEVLRERQENLRLTRLMQDKEEMIGKLKEEIDLLNR   304

Human     RDANVSGTLVSSST-LEKKIEDLEKEVVRERQENLRLVRLMQDKEEMIGKLKEEIDLLNR   312

Chimpanze RDANVSGTLVSSST-LEKKIEDLEKEVVRERQENLRLVRLMQDKEEMIGKLKEEIDLLNR   312

Macaque   RDANVSGNLVSSST-LEKKIEDLEKEVVRERQENLRLVRLMQDKEEMIGKLKEEIDLLNR   312

Cow       RDANASGNLVSSSStLEKKIEDLEKEVVRERQENLRLVRLMQDKEEMIGKLKEEIDLLNR   234

Horse     RDANASGNLLSSST-LEKKIEDLEKEVVRERQENLRLVRLMQDKEEMIGKLKEEIDLLNR   185

Dog       RDANTSGNLVSSSM-LEKKIEDLEKEVVRERQENLRLVRLMQDKEEMIGKLKEEIDLLNR   139

Shrew     XDANSSSNLISRSS-FEKKIEDLEREVVRERQENLRLVRLMQDKEEMIGKLKEEIDLLNK   188

Opossum   RDANSSGNLVPNIT-LEKKIEDLEKEVLKERQENLRLTRLIQDKEETIGKLKEEIDLLNR   311

Chicken   RDANSSGSSVPSNA-LEKRIEELERELAKERQENVRLMKMTQDKEEVIGKLKEEIDLLNR   182

Xenopus_l RETSSSGNSGQDSA-LERKIEELERDLAQERQEHLRLTKIMQDKEELIHKLTEEIDLLNR   319

Zebrafish RDSSDGApta-----LERRVEELERELALEREENERLLKAYQEQDELIMKLKEEIDLLNR   277

Tetradon  REPAPGG--------LDKRMEELEKELARERQENLRLLKAQQDKDDIIRKLREEIDLLNR   305

          =======        =============================================
```

Fig. S6

```
Mouse     DLDDMEDENEQLKQENKTLLKVVGQLTR                                   333

Rat       DLDDMEDENEQLKQENKTLLKVVGQLTR                                   332

Human     DLDDIEDENEQLKQENKTLLKVVGQLTR                                   340

Chimpanze DLDDIEDENEQLKQENKTLLKVVGQLTR                                   340

Macaque   DLDDIEDENEQLKQENKTLLKVVGQLTR                                   340

Cow       DLDDIEDENEQLKQENKTLLKVVGQLTR                                   262

Horse     DLDDIEDENEQLKQENKTLLKVVGQLTR                                   213

Dog       DLDDIEDENEQLKQENKTLLKVVGQLTR                                   167

Shrew     DLGDTENENEKRKQENKTILKVVEQLTR                                   216

Opossum   DLDDIEDENEQLKQENKTLLKVVGQLTR                                   339

Chicken   DLDDIEDENEQLKQENKTLLKVVGQLTR                                   210

Xenopus_l DLDDLEDENKQLKQENKTLLKVVGQLTR                                   347

Zebrafish DLDDIEDENEQLKQENKTLLKVVGQLTR                                   305

Tetradon  DLDDIDDENEQLKQENKTLLKVVGQLTR                                   333

          ============================
```
